# MUC1-C Dictates JUN and BAF-Mediated Chromatin Remodeling at Enhancer Signatures in Cancer Stem Cells

**DOI:** 10.1158/1541-7786.MCR-21-0672

**Published:** 2022-01-12

**Authors:** Atrayee Bhattacharya, Atsushi Fushimi, Nami Yamashita, Masayuki Hagiwara, Yoshihiro Morimoto, Hasan Rajabi, Mark D. Long, Maha Abdulla, Rehan Ahmad, Kelly Street, Song Liu, Tao Liu, Donald Kufe

**Affiliations:** 1 Dana-Farber Cancer Institute, Harvard Medical School, Boston, Massachusetts.; 2 Department of Biostatistics and Bioinformatics, Roswell Park Comprehensive Cancer Center, Buffalo, New York.; 3 Colorectal Research Chair, Department of Surgery, College of Medicine, King Saud University, Riyadh, Saudi Arabia.; 4 Department of Data Science, Dana-Farber Cancer Institute, Department of Biostatistics, Harvard T.H. Chan School of Public Health, Boston, Massachusetts.

## Abstract

The oncogenic MUC1-C protein promotes dedifferentiation of castrate-resistant prostate cancer (CRPC) and triple-negative breast cancer (TNBC) cells. Chromatin remodeling is critical for the cancer stem cell (CSC) state; however, there is no definitive evidence that MUC1-C regulates chromatin accessibility and thereby expression of stemness-associated genes. We demonstrate that MUC1-C drives global changes in chromatin architecture in the dedifferentiation of CRPC and TNBC cells. Our results show that MUC1-C induces differentially accessible regions (DAR) across their genomes, which are significantly associated with differentially expressed genes (DEG). Motif and cistrome analysis further demonstrated MUC1-C–induced DARs align with genes regulated by the JUN/AP-1 family of transcription factors. MUC1-C activates the BAF chromatin remodeling complex, which is recruited by JUN in enhancer selection. In studies of the *NOTCH1* gene, which is required for CRPC and TNBC cell self-renewal, we demonstrate that MUC1-C is necessary for (i) occupancy of JUN and ARID1A/BAF, (ii) increases in H3K27ac and H3K4me3 signals, and (iii) opening of chromatin accessibility on a proximal enhancer–like signature. Studies of the *EGR1* and *LY6E* stemness–associated genes further demonstrate that MUC1-C–induced JUN/ARID1A complexes regulate chromatin accessibility on proximal and distal enhancer–like signatures. These findings uncover a role for MUC1-C in chromatin remodeling that is mediated at least in part by JUN/AP-1 and ARID1A/BAF in association with driving the CSC state.

**Implications:**

These findings show that MUC1-C, which is necessary for the CRPC and TNBC CSC state, activates a novel pathway involving JUN/AP-1 and ARID1A/BAF that regulates chromatin accessibility of stemness-associated gene enhancers.

## Visual Overview

**Figure:**
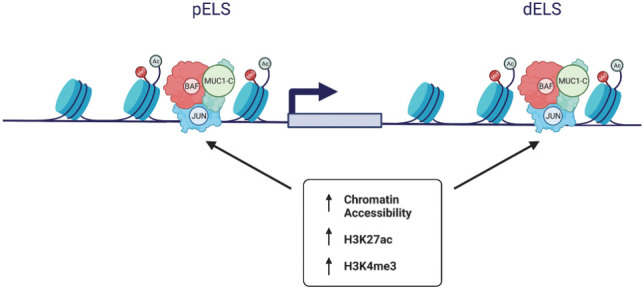

## Introduction

The *MUC1* gene evolved in mammals to protect epithelia from exposure to the external environment (1). The transmembrane MUC1-C subunit is expressed at the apical borders of epithelial cells and is activated by loss of homeostasis associated with infections, inflammation, and physical damage (1). Activated MUC1-C homodimers promote proliferative, inflammatory, and remodeling responses associated with wound healing (1). Prolonged activation of MUC1-C as in chronic inflammation with repetitive cycles of damage and repair contributes to oncogenesis (1, 2). In association with this capacity, MUC1-C is aberrantly expressed in carcinomas, localizes to the nucleus, and drives multiple hallmarks of the cancer cell, including induction of the epithelial-to-mesenchymal transition (EMT) and epigenetic reprogramming (1, 3, 4). MUC1-C also induces DNA methyltransferases (DNMT) and DNA methylation of tumor suppressor genes (TSG; ref. 5). In addition, MUC1-C activates the polycomb repressive complex PRC2, interacts with EZH2, and promotes H3K27 trimethylation in suppressing gene expression (6). The effects of MUC1-C on transcriptomes of cancer cells are not limited to gene repression in that MUC1-C interacts directly with transcription factors, such as (i) MYC in driving dedifferentiation (2, 7, 8) and (ii) E2F1 in activating components of the embryonic stem cell BAF (esBAF) and polybromo BAF (PBAF) chromatin remodeling complexes (9, 10). Whereas MUC1-C controls the activity of these transcription factors, it is not known how MUC1-C contributes to their transactivation functions.

The importance of MUC1-C–induced epigenetic reprogramming and gene regulation has been further supported by the involvement of MUC1-C in driving dedifferentiation in cancer progression (1, 2, 7, 8). MUC1-C promotes the progression of castration-resistant prostate cancer (CRPC) to the highly aggressive form of neuroendocrine prostate cancer (NEPC) by inducing NE dedifferentiation, self-renewal, and tumorigenicity (8). In addition, MUC1-C drives reprogramming and dedifferentiation of triple-negative breast cancer (TNBC) cells (7). These findings have supported involvement of MUC1-C as an effector of the cancer stem cell (CSC) state. Chromatin remodeling is critical to attain the pluripotent state and lineage plasticity (11, 12). However, there has been no evidence linking MUC1-C–induced dedifferentiation with alterations in chromatin accessibility. This work reveals an unrecognized role for MUC1-C in driving global chromatin remodeling in association with the activation of genes that contribute to CSC progression. Our results demonstrate that MUC1-C induces changes in chromatin accessibility at proximal enhancer–like and distal enhancer–like signatures of stemness-associated genes that are mediated by JUN/AP-1 and ARID1A/BAF. There is no previous evidence linking MUC1-C to (i) JUN or ARID1A at enhancer-like signatures, or (ii) their regulation of chromatin accessibility.

## Materials and Methods

### Cell culture

Human DU-145 CRPC cells (ATCC) were cultured in RPMI1640 medium (Thermo Fisher Scientific) containing 10% heat-inactivated FBS (GEMINI Bio-Products). Human BT-549 TNBC cells (ATCC) were cultured in RPMI1640 medium (Thermo Fisher Scientific) containing 10% heat-inactivated FBS (GEMINI Bio-Products), 100 μg/mL streptomycin, 100 U/mL penicillin, and 10 μg/mL insulin. Authentication of the cells was performed by short tandem repeat (STR) analysis. Cells were monitored for *Mycoplasma* contamination using the MycoAlert Mycoplasma Detection Kit (Lonza).

### Gene silencing

MUC1shRNA (MISSION shRNA TRCN0000122938; Sigma) or a control scrambled shRNA (CshRNA; Sigma) was inserted into the pLKO-tet-puro vector (Addgene). JUNshRNA (sc-29223-SH, Santa Cruz Biotechnology), ARID1AshRNA (MISSION shRNA TRCN0000059090), NOTCH1shRNA (MISSION shRNA TRCN0000003362), or a control scrambled shRNA (CshRNA; Sigma) was inserted in the pLKO-puro vector (Addgene RRID:Addgene_32017). The viral vectors were produced in 293T cells (KCB catalog no. KCB 200744YJ, RRID:CVCL_0063; ref. 8). Cells transduced with the vectors were selected for growth in 1–3 μg/mL puromycin. Cells expressing the tet-inducible vectors were treated with 0.1% DMSO as the vehicle control or doxycycline (Millipore Sigma).

### qRT-PCR

Total cellular RNA was isolated using TRIzol reagent (Thermo Fisher Scientific). cDNAs were synthesized and amplified as described previously (8). Primers used for qRT-PCR are listed in Supplementary Table S1.

### Immunoblotting

Total lysates prepared from subconfluent cells were immunoblotted with anti-MUC1-C (HM-1630-P1ABX, 1:400 dilution; Thermo Fisher Scientific), anti-JUN (3742, 1:1,000; Cell Signaling Technology), anti-ARID1A (12354, 1:1,000; Cell Signaling Technology), anti-NOTCH1 (3608, 1:1,000; Cell Signaling Technology), anti-β-actin (A5441, 1:100,000; Sigma), and anti-GAPDH (5174, 1:5,000; Cell Signaling Technology).

### Assay for transposase-accessible chromatin using sequencing

Assay for transposase-accessible chromatin using sequencing (ATAC-seq) libraries were generated from three biologically independent replicates per condition. Library preparation and quality control were performed as described previously (13). Briefly, 50,000 cells were subjected to tagmentation reactions with 2.5 μL Tn5 Transposase (Illumina), purified with Qiagen MinElute PCR Purification Kit (Qiagen) and PCR amplified for 8–9 cycles. Quality of ATAC-seq libraries was confirmed with Bioanalyzer prior to sequencing.

### ATAC-seq analysis

A customized analysis pipeline was applied to process the sequencing data of ATAC-seq libraries (https://github.com/macs3-project/genomics-analysis-pipelines). The data from each replicate were aligned to the human genome (version GRCh38) with bwa-mem (https://arxiv.org/abs/1303.3997v2), evaluated by customized quality control scripts, and cleaned by Picard (https://broadinstitute.github.io/picard/, RRID:SCR_006525) tools to mark and remove the duplicated reads. Detection of the accessible regions for each replicate was performed using MACS2 (14) with q-value cutoff 0.05 in paired-end mode. To combine all replicates, all peaks from replicates were first merged, then only the consensus genomic regions detected as accessible region in at least three replicates regardless the condition were kept, and the regions were split into nonoverlapping 100-bps bins. The UCSC toolkit bigWigAverageOverBed was used to extract the count of fragments in each bin for each replicate and to make a count-table for the differential analysis.

### Chromatin accessibility assay

DNase1 chromatin accessibility assays were performed on chromatin isolated as described previously (15, 16). Aliquots of chromatin were left untreated or digested with 3 U/100 μL DNase I (Promega) for 7 minutes at room temperature. Reactions were stopped by addition of 10 mmol/L EDTA and 2 mmol/L EGTA and incubated at 65°C for 10 minutes. DNA was then treated with RNase (Ambion, Thermo Fisher Scientific) for 30 minutes at 37°C and Proteinase K (Invitrogen, Thermo Fisher Scientific) for 2 hours at 65°C. DNA was purified and amplified by qPCR using primers for the *NOTCH1*, *EGR1*, and *LY6E* genes (Supplementary Table S2). qPCR results were analyzed according to the formula 100/2*^Ct^*^(DNase I) −^*^Ct^*^(no DNase I)^. The data were normalized to input DNA without DNase I treatment.

### Differential accessibility analysis

The count-table containing fragment counts in each 100 bps bin within consensus accessible regions for each replicate was loaded into DESeq2 (RRID:SCR_015687; ref. 17). Then the Wald test for the negative binominal general linear model coefficients was used to call the differential accessible regions (DAR). A fold-change cutoff of 2 and FDR cutoff 1e−5 was applied for DU-145 cells, and FDR cutoff 1e-2 for BT-549 cells. The more stringent cutoff was used for DU-145 to control the total number of differential accessible regions called from both cells to around 10,000. The DARs were further categorized into DARs with increased and decreased accessibilities in response to doxycycline treatment.

### Annotation of DARs

The ChIPseeker package (18) was used to determine the genomic distribution of opening and closing DARs. The “annotatePeak” function was used to find the percentage of DARs located in either promoters, UTRs, exons, introns, downstream, or distal intergenic regions, while defining the promoter regions as 3,000 bps up and downstream from transcription start sites (TSS). Genes with TSSs within 100 kbps of the DARs were identified and then enriched using “enrichGO” function in ClusterProfiler (19) with a Benjamini–Hochberg adjusted FDR cutoff of 0.05, minimum group size of 5, and maximum group size of 500. The Giggle (20) tool was used to query the DARs against the collection of the human ENCODE Candidate Cis-Regulatory Elements (ENCODE cCREs; ref. 21) derived from 706 DNase-seq experiments. The query results contained log2 odds-ratios (ORs) and *P* values from Fisher exact test based on the overlap of DARs and either of the five categories of cCREs, including the pELS, dELS, and PLS. Giggle was also used to search against all publicly available ChIP-seq datasets. The target datasets are the top 1,000 peaks from every one of the 13,976 ChIP-seq datasets collected in the Cistrome DB (22). In addition, the giggle score (combination of *P* value and OR) was used to represent the significance of the overlap in Supplementary Fig. S2.

### Motif analysis of DARs near DEGs

The BETA (RRID:SCR_005396; ref. 23) tool was used to search for enriched motifs in the DARs near the up- or downregulated genes under doxycycline treatment. A *P* value cutoff of 0.05 in the Kolmogorov–Smirnov test was applied to find the list of DEGs close to the DARs. The enrichGO function in clusterProfiler (RRID:SCR_016884; ref. 19) tool was used to identify enriched GO terms in the four categories of genes – upregulated DEGs close to opening DARs, upregulated DEGs close to closing DARs, downregulated DEGs close to opening DARs, and downregulated DEGs close to closing DARs.

### RNA-seq analysis

Total RNA from cells cultured in triplicate was isolated using RNeasy Plus Mini Kit (Qiagen). TruSeq Stranded mRNA (Illumina) was used for library preparation. RNA-seq analysis was performed as described previously (7, 8, 24). The AP-1 Q4 and Q6 gene signatures were obtained from the Molecular Signature Database (MSigDB).

### Chromatin immunoprecipitation

Chromatin immunoprecipitation (ChIP) was performed on cells crosslinked with 1% formaldehyde for 5 minutes at 37°C, quenched with 2 mol/L glycine and washed with PBS, and then sonicated in a Covaris E220 sonicator to generate 300–600 bp DNA fragments. Immunoprecipitation was performed using a control IgG (Santa Cruz Biotechnology) and antibodies against MUC1-C (Cell Signaling Technology, catalog no. 16564, RRID:AB_2798765), JUN (Abcam, catalog no. ab32137, RRID:AB_731608), ARID1A (Cell Signaling Technology, catalog no. 12354, RRID: AB_263710), PBRM1 (Cell Signaling Technology, catalog no. E6N2K), EP300 (Cell Signaling Technology, catalog no. D2X6N), H3K27ac (Abcam, catalog no. ab4729, RRID:AB_2118291), H3K4me1 (Abcam, catalog no. ab8895, RRID:AB_306847), and H3K4me3 (Abcam, catalog no. ab8580; RRID:AB_306649). Precipitated DNAs were detected by PCR using primers listed in Supplementary Table S3. Quantitation was performed on immunoprecipitated DNA using SYBR-green and the CFX384 Real-Time PCR Machine (Bio-Rad). Data are reported as fold enrichment relative to IgG (8).

### Direct binding studies

pMIEG-c-Jun (Addgene plasmid #40348 RRID: Addgene_21915-plko tet puro) was used to construct and purify full-length (FL) GST-JUN (aa 1–331) and to generate the (i) GST-JUN (aa 1–194) and (ii) GST-JUN (aa 195–331) fragments. GST-MUC1-CD (FL; aa 1–72), GST-MUC1-CD (aa 1–45), GST-MUC1-CD (aa 46–72), and GST-MUC1-CD (CQC→AQA) were prepared as described previously (7). GST-JUN and GST-MUC1-CD proteins were cleaved with thrombin to remove GST. Purified JUN was incubated with GST or GST-MUC1-CD proteins bound to glutathione beads, and the adsorbates were analyzed by immunoblotting with anti-JUN. Purified MUC1-CD was incubated with GST or GST-JUN proteins bound to glutathione beads, and the adsorbates were analyzed by immunoblotting with anti–MUC1-CD CD1 antibody (7).

### Tumorsphere formation assays

Single-cell suspensions were cultured in MammoCult Human MediumKit (StemCell Technologies) at a density of 5,000 cells per well of a 6-well ultralow attachment culture plate (Corning) for 10 days. Mammospheres with a diameter >50 μm were counted under an inverted microscope in triplicate wells.

### Statistical analysis

Each experiment was performed at least three times. Data are expressed as the mean ± SD. All statistical analyses for qRT-PCR and ChIP studies were performed by the unpaired Mann–Whitney *U* test or Student *t* test to determine differences between means of groups. A *P* value of < 0.05 denoted by an asterisk (*) was considered statistically significant.

### Data accessibility

The RNA-seq and ATAC-seq data have been deposited in the GEO database under accession codes GSE139335, GSE164141, and GSE180599.

## Results

### Silencing of MUC1-C leads to global changes in chromatin accessibility

MUC1-C drives dedifferentiation and self-renewal of DU-145 CRPC cells (8) and BT-549 TNBC cells (7). However, the mechanisms responsible for these findings remain unknown. Accordingly, we selected these models to investigate the potential involvement of MUC1-C–induced chromatin remodeling in association with driving the CSC state. ATAC-seq was therefore performed on DU-145 and BT-549 cells with MUC1-C silencing (Supplementary Fig. S1A; refs. 7, 8) to assess the effects of MUC1-C on chromatin accessibility. The ATAC-seq libraries had distributions of smaller fragment lengths representing internucleosomal open chromatin regions and larger fragments representing areas spanning nucleosomes (Supplementary Fig. S1B). Read density distribution mapped most of the ATAC-seq peaks to within 1 kb of the TSS, indicating positioning at proximal regions (Supplementary Fig. S1C). Principal components analysis (PCA) across different replicates and doxycycline treatment conditions further showed that PCA sorted according to the amount of data variability and that the samples are clustered by experimental condition (Supplementary Fig. S1D).

**Figure 1.**
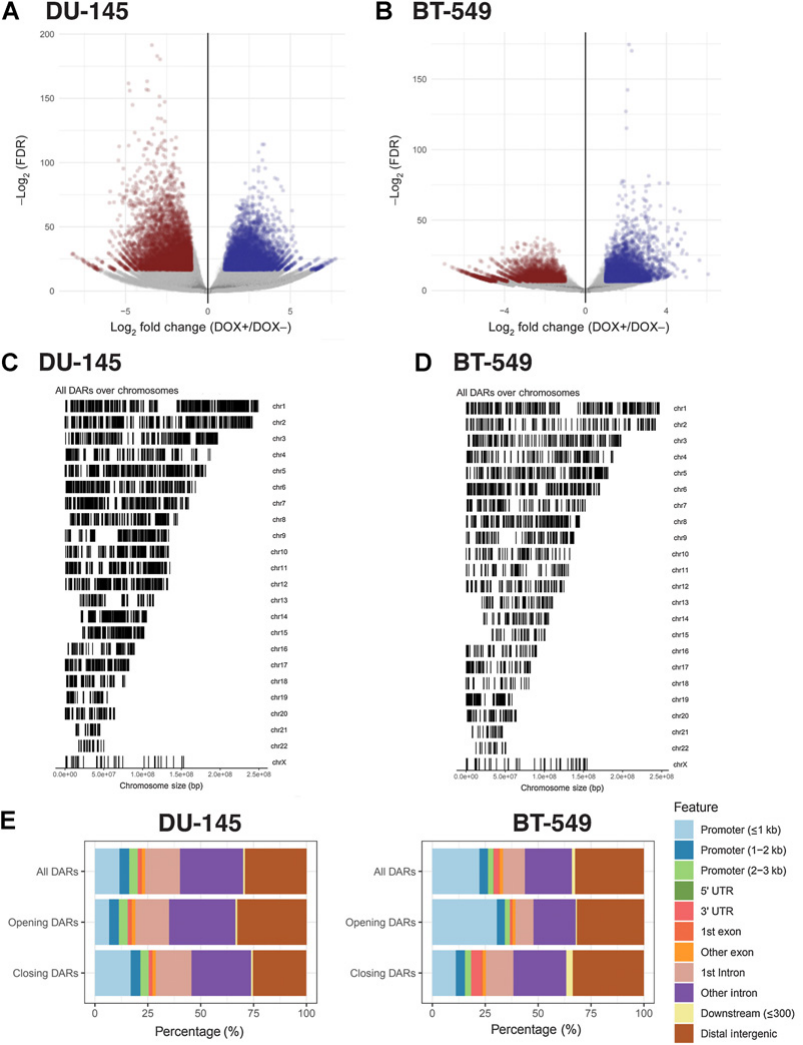
Silencing MUC1-C is associated with global changes in chromatin accessibility. **A** and **B,** ATAC-seq analyses were performed in triplicate on DU-145/tet-MUC1shRNA (**A**) and BT-549/tet-MUC1shRNA (**B**) cells treated with vehicle or doxycycline (DOX) for 7 days. Volcano plots depict DARs with decreased (closing DARs; red) and increased (opening DARs; blue) accessibility as a function of MUC1-C silencing. **C** and **D,** Chromosomal localization of all DARs in MUC1-C–silenced DU-145 (**C**) and BT-549 (**D**) cells. **E,** Genomic distributions of DARs (opening and closing) at the indicated regions in DU-145 (left) and BT-549 (right) cells.

In analyzing the DU-145 cell ATAC-seq samples by DESeq2, we found that silencing MUC1-C results in 12,752 DARs. Of the MUC1-C–regulated DARs, 6,584 and 6,168 were identified with increases and decreases, respectively, in accessibility (Fig. 1A). In BT-549 cell samples, MUC1-C silencing was associated with 8,223 DARs of which 4,730 exhibited increases and 3,493 decreases in accessibility (Fig. 1B). We found that the MUC1-C–induced DARs (all, closing and opening) are widely located over the entire genomes of DU-145 (Fig. 1C; Supplementary Fig. S1E) and BT-549 (Fig. 1D; Supplementary Fig. S1F) cells. In addition, DARs were located at proximal regions within 1–3 kb from the annotated TSS and distal intergenic regions within 50–500 kb from a TSS in DU-145 (Fig. 1E, left) and BT-549 (Fig. 1E, right) cells.

### Association of MUC1-C–induced DARs with biological signaling pathways

**Figure 2.**
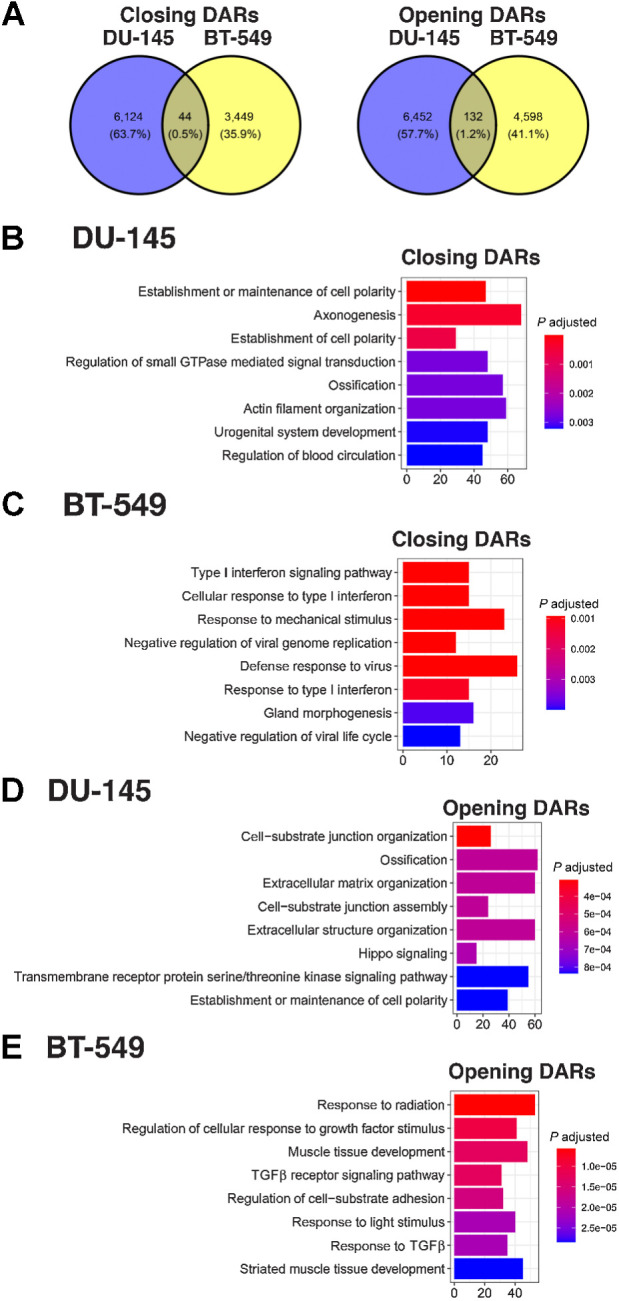
Silencing MUC1-C induces DARs with changes in accessibility that associate with biological processes. **A,** Concordance of closing (left) and opening (right) DARs in MUC1-C–silenced DU-145 and BT-549 cells. **B** and **C,** Associations of closing DARs in DU-145 (**B**) and BT-549 (**C**) cells with the indicated GO BIOLOGICAL PROCESSES. **D** and **E,** Associations of opening DARs in DU-145 (**D**) and BT-549 (**E**) cells with the indicated GO BIOLOGICAL PROCESSES.

Comparison of MUC1-C–induced closing and opening DARs identified 44 and 132, respectively, that are common in DU-145 and BT-549 cells (Fig. 2A). Analysis of the DARs with decreased accessibility for localization to nearest genes and for gene ontology (GO) with ClusterProfiler (19) demonstrated associations with GO BIOLOGICAL PROCESSES of (i) cell polarity, axonogenesis, and actin filament organization in DU-145 cells (Fig. 2B) and (ii) regulation of the type I IFN pathway and response to viral infection in BT-549 cells (Fig. 2C). Of the MUC1-C–induced DARs with increases in accessibility, we identified (i) Hippo and transmembrane receptor serine/threonine kinase signaling pathways in DU-145 cells (Fig. 2D), and (ii) regulation of cellular responses to growth factor stimulus and TGFβ in BT-549 cells (Fig. 2E). We then utilized the Giggle (19) tool to look for potential transcription factor–binding sites or cistromes within the DARs against over 13,000 publicly available human transcription factor ChIP-seq datasets collected in the Cistrome DB (22). We found colocalization of experimentally detected binding sites of JUN and FOS with (i) opening DARs in BT-549 and DU-145 cells, and (ii) closing DARs in BT-549 cells (Supplementary Fig. S2), which was of interest in that there is no known association between MUC1-C and AP-1. NKX2-binding sites were also identified in closing DARs in DU-145 cells (Supplementary Fig. S2).

### Localization of MUC1-C–induced DARs and corresponding DEGs

**Figure 3.**
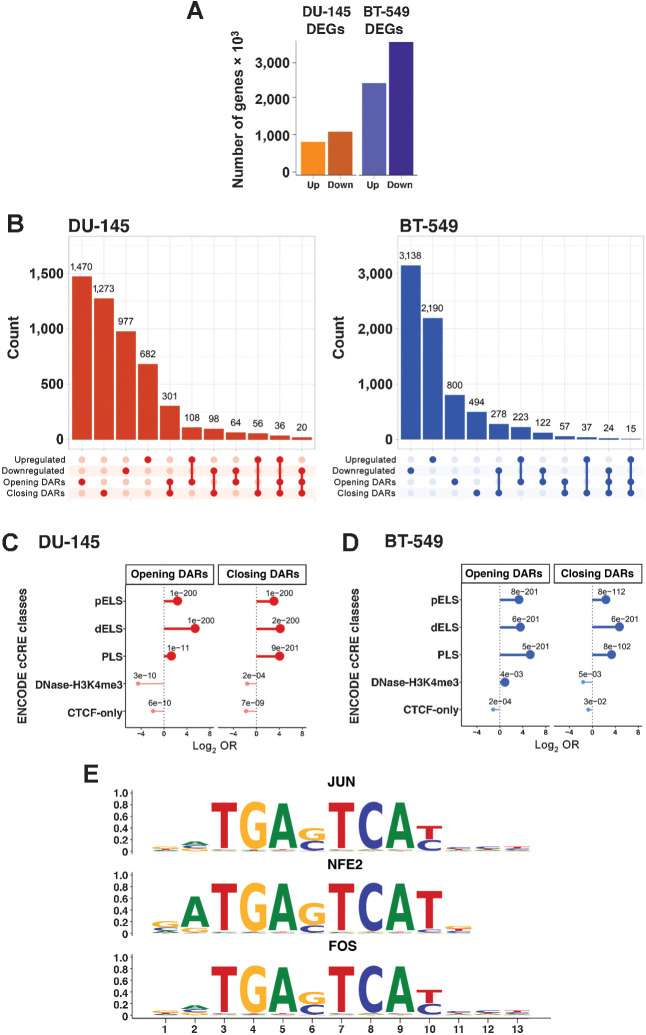
Silencing MUC1-C induces DARs associated with changes in gene expression patterns. **A,** RNA-seq was performed in triplicate on DU-145/tet-MUC1shRNA (left) and BT-549/tet-MUC1shRNA (right) cells treated with vehicle or doxycycline (DOX) for 7 days. Depicted are the numbers of up- and downregulated genes (fold change >1.5 and FDR < 0.05). **B,** Concordance of MUC1-C–induced opening DARs with (1) upregulation and (2) downregulation of genes and closing DARs with (3) upregulation and (4) downregulation of genes in DU-145 (left) and BT-549 (right) cells. **C** and **D,** The lollipop plots showing colocalization of opening and closing DARs at the indicated sites in DU-145 (**C**) and BT-549 (**D**) cells with Candidate *cis*-Regulatory Elements identified by ENCODE (ENCODE cCREs). The *x*-axis shows the fold-change values against genomic background and two-tailed Fisher test *P* values are labeled for the categories with significant enrichment. **E,** Top motifs enriched in DARs near DEGs in DU-145 and BT-549 cells.

RNA-seq demonstrated that MUC1-C silencing in DU-145 cells is associated with 882 upregulated and 1,159 downregulated genes (1.5-fold, FDR<0.05; Fig. 3A, left), and in BT-549 cells with 2,465 upregulated and 3,562 downregulated genes (1.5-fold, FDR<0.05; Fig. 3A, right). In DU-145 cells, comparisons of opening DARs with DEGs identified 144 (108+36) upregulated genes and 84 (64+20) downregulated genes (Fig. 3B, left). Closing DARs were associated with 92 (56+36) upregulated and 118 (98+20) downregulated genes (Fig. 3B, left). Comparisons of opening DARs with DEGs in BT-549 cells further identified 238 (223+15) upregulated genes and 146 (122+24) downregulated genes (Fig. 3B, right). Moreover, closing DARs were associated with 52 (37+15) upregulated and 302 (278+24) downregulated genes (Fig. 3B, right). These findings were extended by demonstrating that MUC1-C–induced DARs and corresponding DEGs significantly correlate with GO BIOLOGICAL PROCESSES associated with stress response and inflammatory signaling pathways (Supplementary Figs. S3A–S3D). In extending this analysis to identify specific TFs that activate these pathways, we found that MUC1-C–induced DARs and corresponding DEGs are located at promoter-like signatures (PLS), proximal enhancer–like signatures (pELS) within 2 kb of a TSS and distal enhancer–like signatures (dELS) that fall more than 2 kb from the nearest TSS (ref. 21; Fig. 3C and D). Consistent with our analysis of ChIP-seq datasets in the Cistrome DB (ref. 22; Supplementary Fig. S2), Binding and Expression Target Analysis (BETA; ref. 23) demonstrated that MUC1-C–induced DARs associated with DEGs are enriched for FOS, JUN, and NEF2-binding motifs (Fig. 3E; Supplementary Fig. S3E, left and right), recognized by members of the activator protein 1 (AP-1) family of leucine zipper TFs.

### MUC1-C–induced DARs and DEGs associate with regulation of AP-1 target gene transcriptomes

**Figure 4.**
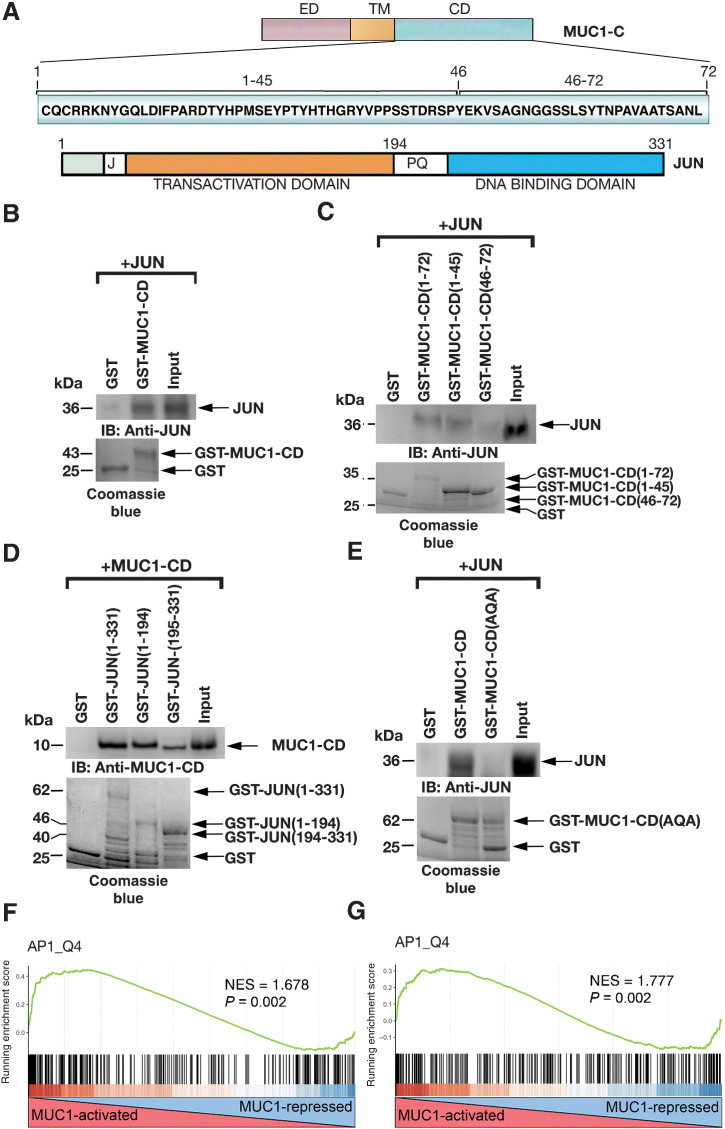
MUC1-C binds directly to JUN and regulates AP-1 target gene signatures. **A,** Schema of the MUC1-C protein with the amino acid sequence of the 72 aa cytoplasmic domain (above). Schema of the JUN 331 aa protein highlighting regions of the transactivation (aa 1-194) and DNA binding (aa 195-331) domains (below). **B,** GST and GST-MUC1-CD (FL; 1-72 aa) were incubated with purified full-length JUN (1-331). **C,** GST, GST-MUC1-CD, GST-MUC1-CD (1–45) and GST-MUC1-CD (45-72) were incubated with purified JUN (1-331). The adsorbates and input were immunoblotted with anti-JUN. Input of the GST proteins was assessed by Coomassie blue staining. **D,** GST, GST-JUN (1-331), GST-JUN (1-194), and GST-JUN(195-331) were incubated with purified MUC1-CD (1-72). The adsorbates and input were immunoblotted with anti-MUC1-CD. **E,** GST, GST-MUC1-CD, and GST-MUC1-CD (mutation of CQC to AQA) were incubated with purified JUN (1-331). The adsorbates and input were immunoblotted with anti-JUN. **F** and **G,** RNA-seq was performed in triplicate on DU-145/tet-MUC1shRNA (**F**) and BT-549/tet-MUC1shRNA (**G**) cells treated with vehicle or doxycycline (DOX) for 7 days. The datasets were analyzed with GSEA using the AP-1 Q4 target gene signature.

Little is known about the involvement of MUC1-C in the regulation of JUN or other AP-1 family members. Accordingly, we focused on JUN and found that MUC1-C has no apparent effect on JUN expression (Supplementary Fig. S4A and S4B). We therefore probed for an interaction between the MUC1-C and JUN proteins. MUC1-C consists of a 58 aa extracellular domain, a 28 aa transmembrane domain, and a 72 aa cytoplasmic domain (Fig. 4A). The MUC1-C cytoplasmic domain (MUC1-CD; 1–72 aa; Fig. 4A) binds directly to certain TFs, such as MYC, and regulates expression of their target genes (7). Along these lines, we found that GST-MUC1-CD (1–72), but not GST, binds directly to full-length JUN (1–331) (Fig. 4B). Further analysis demonstrated that MUC1-CD (1–45) and not MUC1-CD(46–72) binds to JUN (1–331; Fig. 4C). We also found that MUC1-CD binds predominantly to JUN (1–194), which contains the transactivation domain and to a lesser extent with the JUN (195–331) region that includes the DNA-binding domain (Fig. 4D). MUC1-CD includes a CQC motif that is necessary for MUC1-C homodimerization and interactions with certain TFs (1). Mutation of MUC1-CD CQC motif to AQA abrogated binding to JUN in support of dependence on the Cys residues for the interaction (Fig. 4E). In further support of a MUC1-C–JUN interaction, GSEA analysis of the RNA-seq datasets from DU-145 (Fig. 4F; Supplementary Fig. S4C) and BT-549 (Fig. 4G; Supplementary Fig. S4D) cells demonstrated that MUC1-C silencing significantly associates with the regulation of AP-1 target genes. In addition, we found that MUC1-C regulates common sets of AP-1 target genes in DU-145 and BT-549 cells (Supplementary Fig. S4E and S4F), supporting a functional interaction between MUC1-C and JUN in dictating gene expression that is independent of cell context. AP-1 members can recruit BAF to regulate chromatin accessibility (25, 26). ARID1A is an AT-rich interactive domain containing subunit that maintains the BAF complex, particularly at enhancers (27). Along these lines, we found that silencing MUC1 and ARID1A is also associated with the regulation of overlapping sets of AP-1 target genes (Supplementary Fig. S4G and S4H), indicating that ARID1A/BAF may contribute to the regulation of these signatures.

### MUC1-C occupies a *NOTCH1* pELS with JUN and ARID1A in promoting chromatin accessibility

**Figure 5.**
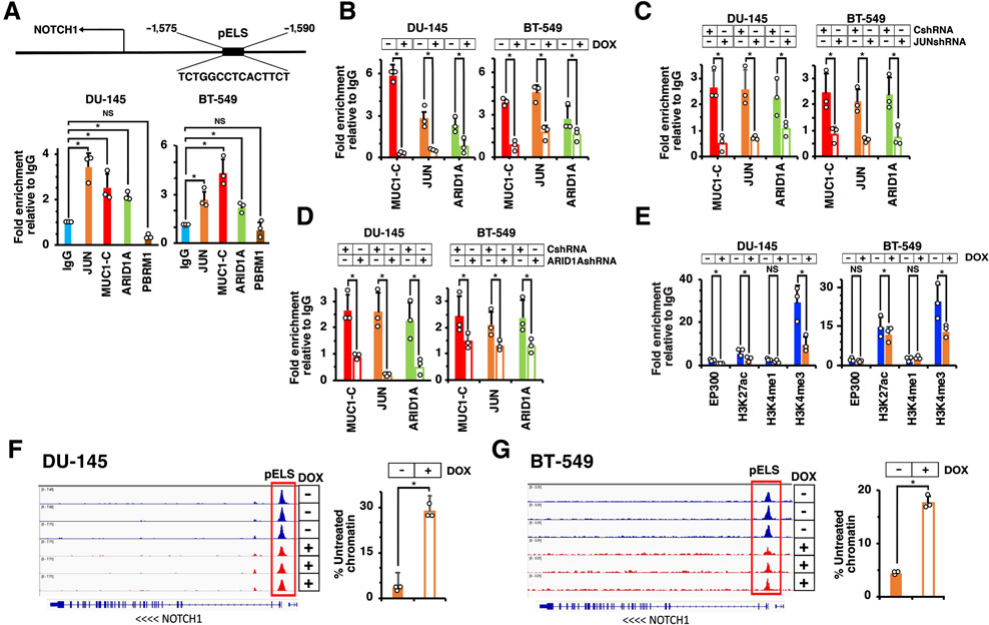
MUC1-C activates the *NOTCH1* pELS region. **A,** Schema of the *NOTCH1* gene with highlighting of the JUN/AP-1 pELS (-1575 to -1590 bp upstream to the TSS). Soluble chromatin from DU-145 (left) and BT-549 (right) cells was precipitated with a control IgG, anti-MUC1-C, anti-JUN, anti-ARID1A, and anti-PBRM1. **B,** Soluble chromatin from DU-145/tet-MUC1shRNA (left) and BT-549/tet-MUC1shRNA (right) cells treated with vehicle or doxycycline (DOX) was precipitated with a control IgG, anti-MUC1-C, anti-JUN, and anti-ARID1A. **C,** Soluble chromatin from DU-145 (left) and BT-549 (right) cells expressing a CshRNA or JUNshRNA was precipitated with a control IgG, anti-MUC1-C, anti-JUN, and anti-ARID1A. **D,** Soluble chromatin from DU-145 (left) and BT-549 (right) cells expressing a CshRNA or ARID1AshRNA was precipitated with a control IgG, anti-MUC1-C, anti-JUN, and anti-ARID1A. **E,** Soluble chromatin from DU-145/tet-MUC1shRNA (left) and BT-549/tet-MUC1shRNA (right) cells treated with vehicle or doxycycline were precipitated with a control IgG, anti-EP300, anti-H3K27ac, anti-H3K4me1, and anti-H3K4me3. The DNA samples were amplified by qPCR with primers for the *NOTCH1* pELS region. The results (mean ± SD of three determinations) are expressed as fold enrichment relative to that obtained with the IgG control (assigned a value of 1). **F** and **G,** Genome browser snapshots of ATAC-seq data from the NOTCH1 pELS in DU-145/tet-MUC1shRNA (**F**) and BT-549/tet-MUC1shRNA (**G**) cells treated with vehicle or doxycycline for 7 days (left). Chromatin was analyzed for accessibility by nuclease digestion (right). The results (mean ± SD of three determinations) are expressed as percent untreated chromatin.

MUC1-C drives NE dedifferentiation of CRPC/NEPC cells by activating the ARID1A/BAF complex and inducing NOTCH1 expression (8, 9). MUC1-C also activates the ARID1A/BAF complex in TNBC cells (9) and, as reported for MUC1-C (4, 7), NOTCH1 plays a role in the progression of TNBC cells (28, 29). In concert with direct binding of MUC1-C and JUN, we detected occupancy of MUC1-C/JUN complexes on the *NOTCH1* gene in a region (-1575 to -1590 bp upstream to the TSS) that contains an AP-1–binding motif (Fig. 5A; Supplementary Fig. S5A). We also identified occupancy of ARID1A and not that of the PBAF PBRM1 subunit (Fig. 5A). Significantly, silencing MUC1-C suppressed JUN and ARID1A occupancy (Fig. 5B). In addition, (i) silencing JUN (Supplementary Fig. S5B) decreased occupancy of MUC1-C and ARID1A (Fig. 5C) and (ii) silencing ARID1A (Supplementary Fig. S5C) was associated with decreases in MUC1-C and JUN occupancy (Fig. 5D). These findings thus supported the identification of a MUC1-C/JUN/ARID1A complex on a *NOTCH1* pELS that is dependent on the presence of each component. Consistent with a pELS (21), we detected EP300 and H3K27ac, as well as relatively higher levels of H3K4me3 compared with H3K4me1 signals, all of which were decreased by MUC1-C silencing (Fig. 5E). In addition, silencing MUC1-C decreased chromatin accessibility as evidenced by the IGV snapshot (30) and by direct nuclease analysis of that region in DU-145 (Fig. 5F, left and right) and BT-549 (Fig. 5G, left and right) cells. As previously shown in DU-145 cells (8, 10), silencing MUC1-C in BT-549 cells downregulated NOTCH1 expression (Supplementary Fig. S5D). In addition, silencing JUN and ARID1A suppressed NOTCH1 expression in DU-145 and BT-549 cells (Supplementary Fig. S5E and S5F). NOTCH1 is necessary for self-renewal of DU-145 cells (9). By extension, silencing NOTCH1 in BT-549 cells was similarly associated with inhibition of tumorsphere formation (Supplementary Fig. S5G and S5H), indicating that MUC1-C–induced regulation of *NOTCH1* chromatin accessibility and expression contributes to the CSC state in different types of carcinoma cells.

### MUC1-C integrates activation of the *EGR1* pELS and dELS

**Figure 6.**
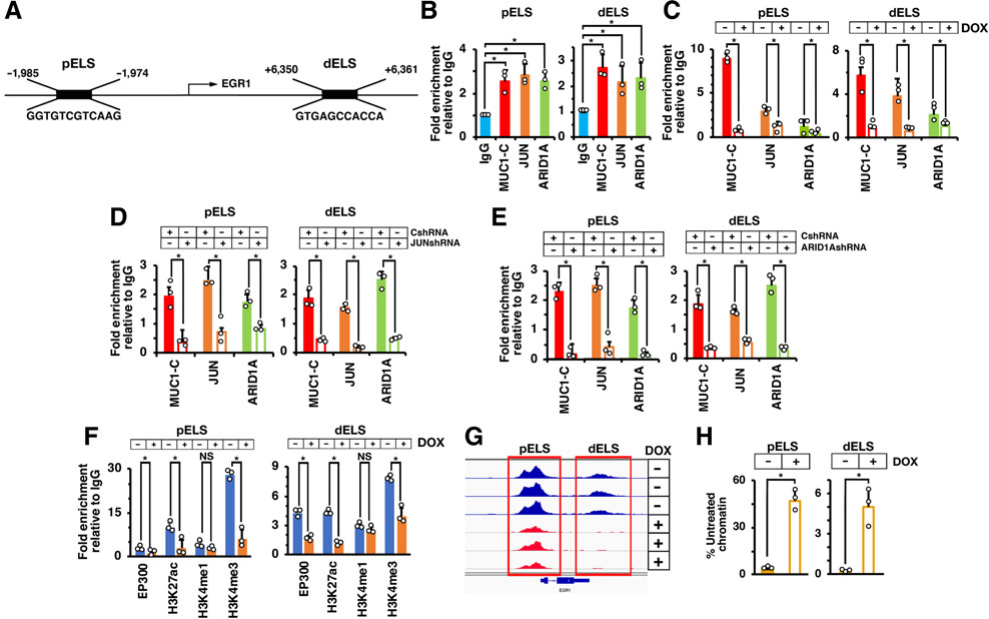
MUC1-C, JUN, and ARID1A occupy the *EGR1* pELS and dELS. **A,** Schema of *EGR1* with highlighting of the pELS and dELS signatures. **B,** Soluble chromatin from DU-145 cells was precipitated with a control IgG, anti-MUC1-C, anti-JUN, anti-ARID1A, and anti-PBRM1. **C,** Soluble chromatin from DU-145/tet-MUC1shRNA cells treated with vehicle or doxycycline (DOX) was precipitated with a control IgG, anti-MUC1-C, anti-JUN, and anti-ARID1A. **D,** Soluble chromatin from DU-145/CshRNA and DU-145/JUNshRNA cells was precipitated with a control IgG, anti-MUC1-C, anti-JUN, and anti-ARID1A. **E,** Soluble chromatin from DU-145/CshRNA and DU-145/ARID1AshRNA cells was precipitated with a control IgG, anti-MUC1-C, anti-JUN, and anti-ARID1A. **F,** Soluble chromatin from DU-145/tet-MUC1shRNA cells treated with vehicle or doxycycline was precipitated with a control IgG, anti-EP300, anti-H3K27ac, anti-H3K4me1, and anti-H3K4me3. The DNA samples were amplified by qPCR with primers for the *EGR1* pELS (left) and dELS (right). The results (mean ± SD of three determinations) are expressed as fold enrichment relative to that obtained with the IgG control (assigned a value of 1). **G** and **H,** DU-145/tet-MUC1shRNA cells were treated with vehicle or doxycycline for 7 days. Genome browser snapshots of ATAC-seq data from the EGR1 pELS and dELS signatures. Chromatin was analyzed for accessibility by nuclease digestion (**H**). The results (mean ± SD of three determinations) are expressed as percent untreated chromatin.

Among the common sets of MUC1-C–induced AP-1 target genes, we identified *EGR1*, which is associated with promoting CRPC and TNBC CSC states (31–33). Having demonstrated that MUC1-C regulates chromatin accessibility of the *NOTCH1* pELS, *EGR1* was of interest in that putative AP-1–binding elements were identified in a (i) potential pELS (-1985 to -1974 bp) within 2 kb from the TSS, and (ii) dELS (+6350 to +6361) at over 6 kb from the TSS (Fig. 6A). We found that MUC1-C, JUN, and ARID1A occupy the *EGR1* pELSs and dELSs in DU-145 (Fig. 6B, left and right) and BT-549 (Supplementary Fig. S6A, left and right) cells. Moreover, as identified in the *NOTCH1* studies, silencing MUC1-C (Fig. 6C, left and right; Supplementary Fig. S6B, left and right), JUN (Fig. 6D, left and right; Supplementary Fig. S6C, left and right) or ARID1A (Fig. 6E, left and right; Supplementary Fig. S6D, left and right) decreased occupancy the MUC1-C/JUN/ARID1A complex on the *EGR1* pELSs and dELSs. In addition, we found that silencing MUC1-C in DU-145 cells decreased (i) EP300 occupancy, and (ii) H3K27ac and H3K4me3 signals on the *EGR1* pELS and dELS (Fig. 6F, left and right). In BT-549 cells, similar results were obtained for the *EGR1* pELS; however, there was no enrichment of H3K27ac or H3K4me3 at the *EGR1* dELS (Supplementary Fig. S6E, left and right), suggesting that the *EGR1* dELS may be an inactive site. Nonetheless, chromatin accessibility of the *EGR1* pELS and dELS was decreased by silencing MUC1-C in DU-145 (Fig. 6G and H) and BT-549 (Supplementary Fig. S6F and S6G) cells. Consistent with these results, silencing MUC1-C, JUN, and ARID1A also resulted in suppression of EGR1 expression in DU-145 and BT-549 cells (Supplementary Figs. S6H–S6J).

### MUC1-C differentially activates *LY6E* pELSs and dELSs based on cell context

**Figure 7.**
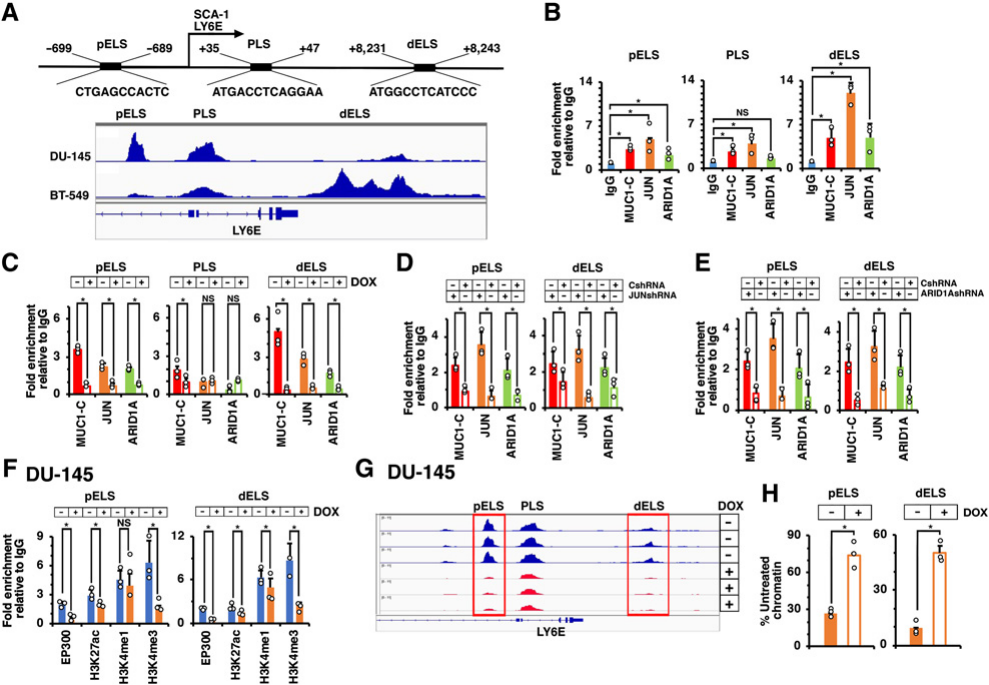
MUC1-C integrates activation of the *LY6E* pELS and dELS regions. **A,** Schema of *LY6E* with highlighting of JUN/AP-1–binding sites and chromatin accessibility of the pELS, PLS, and dELS signatures in DU-145 and BT-549 cells. **B,** Soluble chromatin from DU-145 cells was precipitated with a control IgG, anti-MUC1-C, anti-JUN, anti-ARID1A, and anti-PBRM1. **C,** Soluble chromatin from DU-145/tet-MUC1shRNA cells treated with vehicle or doxycycline (DOX) was precipitated with a control IgG, anti-MUC1-C, anti-JUN, and anti-ARID1A. The DNA samples were amplified by qPCR with primers for the *LY6E* pELS (left), PLS (middle), and dELS (right) regions. **D,** Soluble chromatin from DU-145/CshRNA and DU-145/JUNshRNA cells was precipitated with a control IgG, anti-MUC1-C, anti-JUN, and anti-ARID1A. **E,** Soluble chromatin from DU-145/CshRNA and DU-145/ARID1AshRNA cells was precipitated with a control IgG, anti-MUC1-C, anti-JUN, and anti-ARID1A. **F,** Soluble chromatin from DU-145/tet-MUC1shRNA cells treated with vehicle or doxycycline was precipitated with a control IgG, anti-EP300, anti-H3K27ac, anti-H3K4me1, and anti-H3K4me3. The DNA samples were amplified by qPCR with primers for the *LY6E* pELS (left) and dELS (right) regions. The results (mean ± SD of three determinations) are expressed as fold enrichment relative to that obtained with the IgG control (assigned a value of 1). **G** and **H,** DU-145/tet-MUC1shRNA cells were treated with vehicle or doxycycline for 7 days. Genome browser snapshots of ATAC-seq data from the *LY6E* pELS, PLS and dELS (**G**). Chromatin from the pELS and dELS was analyzed for accessibility by nuclease digestion (**H**). The results (mean ± SD of three determinations) are expressed as percent untreated chromatin.

To further investigate involvement of MUC1-C in integrating activation of enhancer regions, we studied the human *LY6E* gene, which encodes a protein related to mouse stem cell factor 1 (Sca-1) and is linked to CRPC and TNBC progression (34–37). Putative AP-1–binding motifs were identified in potential pELS (-699 to -689 bp), PLS (+35 to +47 bp), and dELS (+8231 to +8243 bp), which were of interest in that they exhibited distinct patterns of chromatin accessibility in DU-145 and BT-549 cells (Fig. 7A). In this regard, whereas peaks of chromatin accessible regions were detectable in the PLS in both cell types, the pELS region was more prominent in DU-145 cells and the dELS region was substantially broader and more pronounced in BT-549 cells (Fig. 7A). We found that the pELS and dELS, but not PLS, were significantly occupied by MUC1-C, JUN, and ARID1A in DU-145 cells (Fig. 7B). In BT-549 cells, we detected significant occupancy of MUC1-C, JUN, and ARID1A in the PLS and dELS, but not in the pELS (Supplementary Fig. S7A), conceivably due to the relatively low level of chromatin accessibility in that region. Silencing MUC1-C in DU-145 cells decreased JUN and ARID1A occupancy in the *LY6E* pELS and dELS, but not significantly in the PLS (Fig. 7C). Accordingly, we focused on the *LY6E* pELS and dELS and found that JUN (Fig. 7D) and ARID1A (Fig. 7E) are necessary for occupancy of MUC1-C/JUN/ARID1A complexes. Moreover, in BT-549 cells, we found that MUC1-C (Supplementary Fig. S7B), JUN (Supplementary Fig. S7C), and ARID1A (Supplementary Fig. S7D) are necessary for occupancy of MUC1-C/JUN/ARID1A complexes on the PLS and dELS. Consistent with these results, MUC1-C induced increases in EP300, H3K27ac, and H3K4me3 were detectable in the (i) pELS and dELS in DU-145 cells (Fig. 7F), and (ii) in the PLS in BT-549 cells (Supplementary Fig. S7E). In addition, silencing MUC1-C was associated with decreases in chromatin accessibility and nuclease sensitivity in the pELS and dELS in DU-145 cells (Fig. 7G and H) and in the PLS and dELS in BT-549 cells (Supplementary Figs. S7F and S7G). By extension, MUC1-C, JUN, and ARID1A were each necessary for *LY6E* expression in DU-145 and BT-549 cells (Supplementary Figs. S7H–S7J). These results indicate that MUC1-C–mediated activation the *LY6E* pELS, PLS, and dELS is differentially regulated depending on cell context.

## Discussion

Epigenetic regulatory mechanisms, including ATP-dependent chromatin remodeling, are essential for maintaining tissue homeostasis and the wound healing response (38). The MUC1-C protein promotes activation of inflammatory, proliferative, and repair pathways found in wound healing and cancer (1). MUC1-C induces changes in DNA methylation patterns and histone modifications in cancer cells (1); however, there has been no evidence that MUC1-C dictates chromatin remodeling. This work demonstrates that MUC1-C regulates global remodeling of chromatin accessibility in DU-145 CRPC and BT-549 TNBC cells. We selected these cells based on findings that MUC1-C drives their dedifferentiation and progression (7, 8). These models were also selected to compare effects of MUC1-C on CSCs derived from different tumor types. In this regard, we found that MUC1-C governs similar patterns of global chromatin remodeling in DU-145 and BT-549 cells; that is, induction of DARs across their entire genomes. Subsets of MUC1-C–driven opening and closing DARs were common to both cell types and were distributed at proximal promoter and distal intergenic regions. In addition, most DARs were significantly associated with induction or repression of genes. Parallels in the CRPC and TNBC CSC models were extended by identifying widespread enrichment of binding motifs in publicly available cistromes for the JUN, FOS, and NFE2 TFs and other members of the AP-1 family. AP-1 is a dimeric complex that includes the JUN, FOS, ATF and MAF proteins that form diverse homodimers and heterodimers in regulating oncogenic and tumor-suppressive activities (39, 40). Given these complexities, we focused on JUN with the understanding that MUC1-C may interact with other AP-1 family members. Indeed, little had been known to date about interactions between MUC1-C and AP-1 TFs; in this regard, our findings identified a previously unrecognized MUC1-C–driven program of chromatin regulation.

MUC1-C induces expression of the Yamanaka OCT4, SOX2, KLF4, and MYC (OSKM) pioneer transcription factors in the dedifferentiation of CRPC and TNBC cells (7, 8). Among the OSKM factors, MUC1-C binds directly to MYC in the regulation of MYC target genes (7, 8). The OSKM factors induce dynamic changes in chromatin states during induction of pluripotency and interact with JUN and other AP-1 family members in regulating accessibility (41, 42). We found that MUC1-C binds directly to JUN and forms a nuclear complex with JUN in driving gene expression. In addition, analysis of DU-145 and BT-549 RNA-seq datasets demonstrated that silencing MUC1-C is significantly associated with the activation and repression of common sets of AP-1 target genes. The parallels in the DU-145 and BT-549 cell models were further extended by the demonstration that MUC1-C and JUN are necessary for activation the *NOTCH1* gene, which is of importance for the progression of CSCs (9, 28, 29). MUC1-C/JUN complexes were detectable on a *NOTCH1* pELS, invoking the potential recruitment of the ARID1A/BAF chromain remodeling complex. Along these lines, MUC1-C activates the ARID1A component of BAF in the dedifferentiation of DU-145 and BT-549 cells (9). In addition, AP-1 members can cooperate with cell type–specific TFs in the recruitment of BAF to regulate chromatin accessibility (25, 26). In support of a model in which MUC1-C interacts with JUN with the recruitment of ARID1A to the *NOTCH1* pELS, MUC1-C was necessary for (i) occupancy of JUN and ARID1A complexes, (ii) increases in H3K27ac and H3K4me3 levels, and (iii) opening of chromatin accessibility. The importance of JUN and BAF in this setting was further supported by the dependence on ARID1A for *NOTCH1* activation (9) and the findings that MUC1-C and ARID1A regulate common sets of AP-1 target genes.

Our studies of the *NOTCH1* pELS revealed an unexpected role for MUC1-C in chromatin remodeling and provided the basis for extending these investigations to dELSs. Along these lines, AP-1 can operate both proximally to the TSS and at distal enhancer sites (40, 43). Given the localization MUC1-C–induced DARs to distal intergenic regions, we studied *EGR1* and *LY6E* as other stemness-associated genes with potential pELSs and dELSs. As found for the *NOTCH1* pELS, we detected MUC1-C/JUN/ARID1A complexes occupying the *EGR1* pELS and dELS in DU-145 and BT-549 cells. In addition, silencing MUC1-C, JUN, or ARID1A disrupted occupancy of these complexes, in support of their interdependency for inducing gene activation. MUC1-C also induced increases in chromatin accessibility and DNase sensitivity at the *EGR1* pELS and dELS in both DU-145 and BT-549 cells. Notably, MUC1-C increased H3K27ac and H3K4me3 signals at the *EGR1* pELS and dELS in DU-145 cells and, in contrast, only at the *EGR1* pELS in BT-549 cells, highlighting differences in *EGR1* regulation. As further evidence for cell context–dependent regulation, studies of the *LY6E* gene identified pELSs and dELSs in both DU-145 and BT-549 cells that were distinct in terms of MUC1-C–induced (i) patterns of JUN and ARID1A occupancy, and (ii) intensities of H3K27ac and H3K4me3 signals. MUC1-C was also necessary for driving chromatin accessibility of the pELS and dELS in both cell types. A common finding in our studies of the *NOTCH1*, *EGR1*, and *LY6E* genes is that, in addition to activating H3K27ac signals, MUC1-C was associated with increases in H3K4me3 as compared with H3K4me1. H3K4me3 is a characteristic signal of active promoters and is also detectable at active enhancers bound by RNA Pol II (44, 45). Our work shows that MUC1-C–induced activation of *NOTCH1*, *EGR1*, and *LY6E* is associated with predominant H3K4me3 signals at enhancer-like signatures. Further studies will be needed to address the mechanistic basis for these findings and whether they are related to MUC1-C–induced regulation of H3K4 methyltransferases or demethylases that contribute to aberrantly increased transcriptional activity.

The identification of MUC1-C–induced *EGR1* and *LY6E* stemness-associated genes informs new avenues for investigation of how they may contribute to the CSC state, which will be a focus of our subsequent studies. Additional studies will also be needed to address the diversity of MUC1-C–driven AP-1 target genes, particularly in the context of lineage-specific TFs, which can dictate cell type–specific recruitment of BAF. Along these lines, the observations that silencing MUC1-C and ARID1A is associated with the regulation of common sets of AP-1 target genes indicate that MUC1-C may broadly induce global remodeling of chromatin through AP-1 and the esBAF complex in driving stemness (9). CSCs have the capacity for self-renewal and differentiation. As such, it will be of interest to determine whether changes in CSC chromatin structure are incorporated in their differentiated progeny. Finally, these results do not exclude the possibility that MUC1-C–induced changes in chromatin accessibility are restricted to the BAF complex. Consistent with this notion, MUC1-C activates the NuRD nucleosome and PBAF chromatin remodeling complexes in CRPC and TNBC cells (7, 10), which could further contribute to the effects of MUC1-C on global chromatin architecture.

## Supplementary Material

Supplementary Figure

## References

[1] Kufe D . MUC1-C in chronic inflammation and carcinogenesis; emergence as a target for cancer treatment. Carcinogenesis 2020;41:1173–83. 32710608 10.1093/carcin/bgaa082 PMC7513951

[2] Li W , Zhang N, Jin C, Long MD, Rajabi H, Yasumizu Y, . MUC1-C drives stemness in progression of colitis to colorectal cancer. JCI Insight 2020;5:137112. 32427590 10.1172/jci.insight.137112 PMC7406273

[3] Rajabi H , Hiraki M, Kufe D. MUC1-C activates polycomb repressive complexes and downregulates tumor suppressor genes in human cancer cells. Oncogene 2018;37:2079–88. 29379165 10.1038/s41388-017-0096-9 PMC5908737

[4] Hata T , Rajabi H, Yamamoto M, Jin C, Ahmad R, Zhang Y, . Targeting MUC1-C inhibits TWIST1 signaling in triple-negative breast cancer. Mol Cancer Ther 2019;18:1744–54. 31308076 10.1158/1535-7163.MCT-19-0156 PMC6774902

[5] Rajabi H , Tagde A, Alam M, Bouillez A, Pitroda S, Suzuki Y, . DNA methylation by DNMT1 and DNMT3b methyltransferases is driven by the MUC1-C oncoprotein in human carcinoma cells. Oncogene 2016;35:6439–45. 27212035 10.1038/onc.2016.180 PMC5121097

[6] Rajabi H , Hiraki M, Tagde A, Alam M, Bouillez A, Christensen CL, . MUC1-C activates EZH2 expression and function in human cancer cells. Sci Rep 2017;7:7481. 28785086 10.1038/s41598-017-07850-0 PMC5547076

[7] Hata T , Rajabi H, Takahashi H, Yasumizu Y, Li W, Jin C, . MUC1-C activates the NuRD complex to drive dedifferentiation of triple-negative breast cancer cells. Cancer Res 2019;79:5711–22. 31519689 10.1158/0008-5472.CAN-19-1034 PMC6881519

[8] Yasumizu Y , Rajabi H, Jin C, Hata T, Pitroda S, Long MD, . MUC1-C drives lineage plasticity in progression to neuroendocrine prostate cancer. Nat Commun 2020;11:338. 31953400 10.1038/s41467-019-14219-6 PMC6969104

[9] Hagiwara M , Yasumizu Y, Yamashita N, Rajabi H, Fushimi A, Long MD, . MUC1-C activates the BAF (mSWI/SNF) complex in prostate cancer stem cells. Cancer Res 2021;81:1111–22. 33323379 10.1158/0008-5472.CAN-20-2588 PMC8026569

[10] Hagiwara M , Fushimi A, Yamashita N, Battacharya A, Rajabi H, Long M, . MUC1-C activates the PBAF chromatin remodeling complex in integrating redox balance with progression of human prostate cancer stem cells. Oncogene 2021;40:4920–40. 10.1038/s41388-021-01899-y PMC8321896 34163028

[11] Koche RP , Smith ZD, Adli M, Gu H, Ku M, Gnirke A, . Reprogramming factor expression initiates widespread targeted chromatin remodeling. Cell Stem Cell 2011;8:96–105. 21211784 10.1016/j.stem.2010.12.001 PMC3220622

[12] Polo JM , Anderssen E, Walsh RM, Schwarz BA, Nefzger CM, Lim SM, . A molecular roadmap of reprogramming somatic cells into iPS cells. Cell 2012;151:1617–32. 23260147 10.1016/j.cell.2012.11.039 PMC3608203

[13] Buenrostro JD , Wu B, Chang HY, Greenleaf WJ. ATAC-seq: a method for assaying chromatin accessibility genome-wide. Curr Protoc Mol Biol 2015;109:1–9. 10.1002/0471142727.mb2129s109 PMC4374986 25559105

[14] Zhang Y , Liu T, Meyer CA, Eeckhoute J, Johnson DS, Bernstein BE, . Model-based analysis of ChIP-Seq (MACS). Genome Biol 2008;9:R137. 18798982 10.1186/gb-2008-9-9-r137 PMC2592715

[15] Nepon-Sixt BS , Alexandrow MG. DNase I chromatin accessibility analysis. Bio Protoc 2019;9:e3444. 10.21769/BioProtoc.3444 PMC7854364 33654939

[16] Nepon-Sixt BS , Bryant VL, Alexandrow MG. Myc-driven chromatin accessibility regulates Cdc45 assembly into CMG helicases. Commun Biol 2019;2:110. 30911685 10.1038/s42003-019-0353-2 PMC6430796

[17] Love MI , Huber W, Anders S. Moderated estimation of fold change and dispersion for RNA-seq data with DESeq2. Genome Biol 2014;15:550. 25516281 10.1186/s13059-014-0550-8 PMC4302049

[18] Yu G , Wang LG, He QY. ChIPseeker: an R/Bioconductor package for ChIP peak annotation, comparison and visualization. Bioinformatics 2015;31:2382–3. 25765347 10.1093/bioinformatics/btv145

[19] Yu G , Wang LG, Han Y, He QY. clusterProfiler: an R package for comparing biological themes among gene clusters. OMICS 2012;16:284–7. 22455463 10.1089/omi.2011.0118 PMC3339379

[20] Layer RM , Pedersen BS, DiSera T, Marth GT, Gertz J, Quinlan AR. GIGGLE: a search engine for large-scale integrated genome analysis. Nat Methods 2018;15:123–6. 29309061 10.1038/nmeth.4556 PMC5872823

[21] ENCODE Project Consortium, Moore JE, Purcaro MJ, Pratt HE, Epstein CB, Shoresh N, . Expanded encyclopaedias of DNA elements in the human and mouse genomes. Nature 2020;583:699–710. 32728249 10.1038/s41586-020-2493-4 PMC7410828

[22] Zheng R , Wan C, Mei S, Qin Q, Wu Q, Sun H, . Cistrome data browser: expanded datasets and new tools for gene regulatory analysis. Nucleic Acids Res 2019;47:D729–D35. 30462313 10.1093/nar/gky1094 PMC6324081

[23] Wang S , Sun H, Ma J, Zang C, Wang C, Wang J, . Target analysis by integration of transcriptome and ChIP-seq data with BETA. Nat Protoc 2013;8:2502–15. 24263090 10.1038/nprot.2013.150 PMC4135175

[24] Yamashita N , Long M, Fushimi A, Yamamoto M, Hata T, Hagiwara M, . MUC1-C integrates activation of the IFN-gamma pathway with suppression of the tumor immune microenvironment in triple-negative breast cancer. J Immunother Cancer 2021;9:e002115. 33495298 10.1136/jitc-2020-002115 PMC7839859

[25] Vierbuchen T , Ling E, Cowley CJ, Couch CH, Wang X, Harmin DA, . AP-1 transcription factors and the BAF complex mediate signal-dependent enhancer selection. Mol Cell 2017;68:1067–822. 29272704 10.1016/j.molcel.2017.11.026 PMC5744881

[26] Sen M , Wang X, Hamdan FH, Rapp J, Eggert J, Kosinsky RL, . ARID1A facilitates KRAS signaling-regulated enhancer activity in an AP1-dependent manner in colorectal cancer cells. Clin Epigenetics 2019;11:92. 31217031 10.1186/s13148-019-0690-5 PMC6585056

[27] Mathur R , Alver BH, Roman AKS, Wilson BG, Wang X, Agoston AT, . ARID1A loss impairs enhancer-mediated gene regulation and drives colon cancer in mice. Nat Genet 2017;49:296–302. 27941798 10.1038/ng.3744 PMC5285448

[28] Xie X , Kaoud TS, Edupuganti R, Zhang T, Kogawa T, Zhao Y, . c-Jun N-terminal kinase promotes stem cell phenotype in triple-negative breast cancer through upregulation of Notch1 via activation of c-Jun. Oncogene 2017;36:2599–608. 27941886 10.1038/onc.2016.417 PMC6116358

[29] Miao K , Lei JH, Valecha MV, Zhang A, Xu J, Wang L, . NOTCH1 activation compensates BRCA1 deficiency and promotes triple-negative breast cancer formation. Nat Commun 2020;11:3256. 32591500 10.1038/s41467-020-16936-9 PMC7320176

[30] Robinson JT , Thorvaldsdottir H, Winckler W, Guttman M, Lander ES, Getz G, . Integrative genomics viewer. Nat Biotechnol 2011;29:24–6. 21221095 10.1038/nbt.1754 PMC3346182

[31] Tang T , Zhu Q, Li X, Zhu G, Deng S, Wang Y, . Protease Nexin I is a feedback regulator of EGF/PKC/MAPK/EGR1 signaling in breast cancer cells metastasis and stemness. Cell Death Dis 2019;10:649. 31501409 10.1038/s41419-019-1882-9 PMC6733841

[32] Jacob S , Nayak S, Kakar R, Chaudhari UK, Joshi D, Vundinti BR, . A triad of telomerase, androgen receptor and early growth response 1 in prostate cancer cells. Cancer Biol Ther 2016;17:439–48. 27003515 10.1080/15384047.2016.1156255 PMC4910915

[33] Li L , Ameri AH, Wang S, Jansson KH, Casey OM, Yang Q, . EGR1 regulates angiogenic and osteoclastogenic factors in prostate cancer and promotes metastasis. Oncogene 2019;38:6241–55. 31312026 10.1038/s41388-019-0873-8 PMC6715537

[34] AlHossiny M , Luo L, Frazier WR, Steiner N, Gusev Y, Kallakury B, . Ly6E/K signaling to TGFbeta promotes breast cancer progression, immune escape, and drug resistance. Cancer Res 2016;76:3376–86. 27197181 10.1158/0008-5472.CAN-15-2654 PMC4910623

[35] Luo L , McGarvey P, Madhavan S, Kumar R, Gusev Y, Upadhyay G. Distinct lymphocyte antigens 6 (Ly6) family members Ly6D, Ly6E, Ly6K and Ly6H drive tumorigenesis and clinical outcome. Oncotarget 2016;7:11165–93. 26862846 10.18632/oncotarget.7163 PMC4905465

[36] Barros-Silva JD , Linn DE, Steiner I, Guo G, Ali A, Pakula H, . Single-cell analysis identifies LY6D as a marker linking castration-resistant prostate luminal cells to prostate progenitors and cancer. Cell Rep 2018;25:3504–18. 30566873 10.1016/j.celrep.2018.11.069 PMC6315111

[37] Castagnoli L , Cancila V, Cordoba-Romero SL, Faraci S, Talarico G, Belmonte B, . WNT signaling modulates PD-L1 expression in the stem cell compartment of triple-negative breast cancer. Oncogene 2019;38:4047–60. 30705400 10.1038/s41388-019-0700-2 PMC6755989

[38] Ge Y , Fuchs E. Stretching the limits: from homeostasis to stem cell plasticity in wound healing and cancer. Nat Rev Genet 2018;19:311–25. 29479084 10.1038/nrg.2018.9 PMC6301069

[39] Eferl R , Wagner EF. AP-1: a double-edged sword in tumorigenesis. Nat Rev Cancer 2003;3:859–68. 14668816 10.1038/nrc1209

[40] Bejjani F , Evanno E, Zibara K, Piechaczyk M, Jariel-Encontre I. The AP-1 transcriptional complex: local switch or remote command? Biochim Biophys Acta Rev Cancer 2019;1872:11–23. 31034924 10.1016/j.bbcan.2019.04.003

[41] Li D , Liu J, Yang X, Zhou C, Guo J, Wu C, . Chromatin accessibility dynamics during iPSC reprogramming. Cell Stem Cell 2017;21:819–33. 29220666 10.1016/j.stem.2017.10.012

[42] Chronis C , Fiziev P, Papp B, Butz S, Bonora G, Sabri S, . Cooperative binding of transcription factors orchestrates reprogramming. Cell 2017;168:442–59. 28111071 10.1016/j.cell.2016.12.016 PMC5302508

[43] Madrigal P , Alasoo K. AP-1 takes centre stage in enhancer chromatin dynamics. Trends Cell Biol 2018;28:509–11. 29778529 10.1016/j.tcb.2018.04.009

[44] Heintzman ND , Stuart RK, Hon G, Fu Y, Ching CW, Hawkins RD, . Distinct and predictive chromatin signatures of transcriptional promoters and enhancers in the human genome. Nat Genet 2007;39:311–8. 17277777 10.1038/ng1966

[45] Calo E , Wysocka J. Modification of enhancer chromatin: what, how, and why? Mol Cell 2013;49:825–37. 23473601 10.1016/j.molcel.2013.01.038 PMC3857148

