# Supplementary material for: MUC1-C Dictates JUN and BAF-Mediated Chromatin Remodeling at Enhancer Signatures in Cancer Stem Cells
Source: Mol Cancer Res. 2022 Jan 12;20(4):556–67. doi: 10.1158/1541-7786.MCR-21-0672 (PMC8983489; doi:10.1158/1541-7786.MCR-21-0672)
Supplement: Supplementary Figure [file mcr-21-0672_supplementary_figures_s1-s7_and_tables_s1-s3_supp3.pdf]

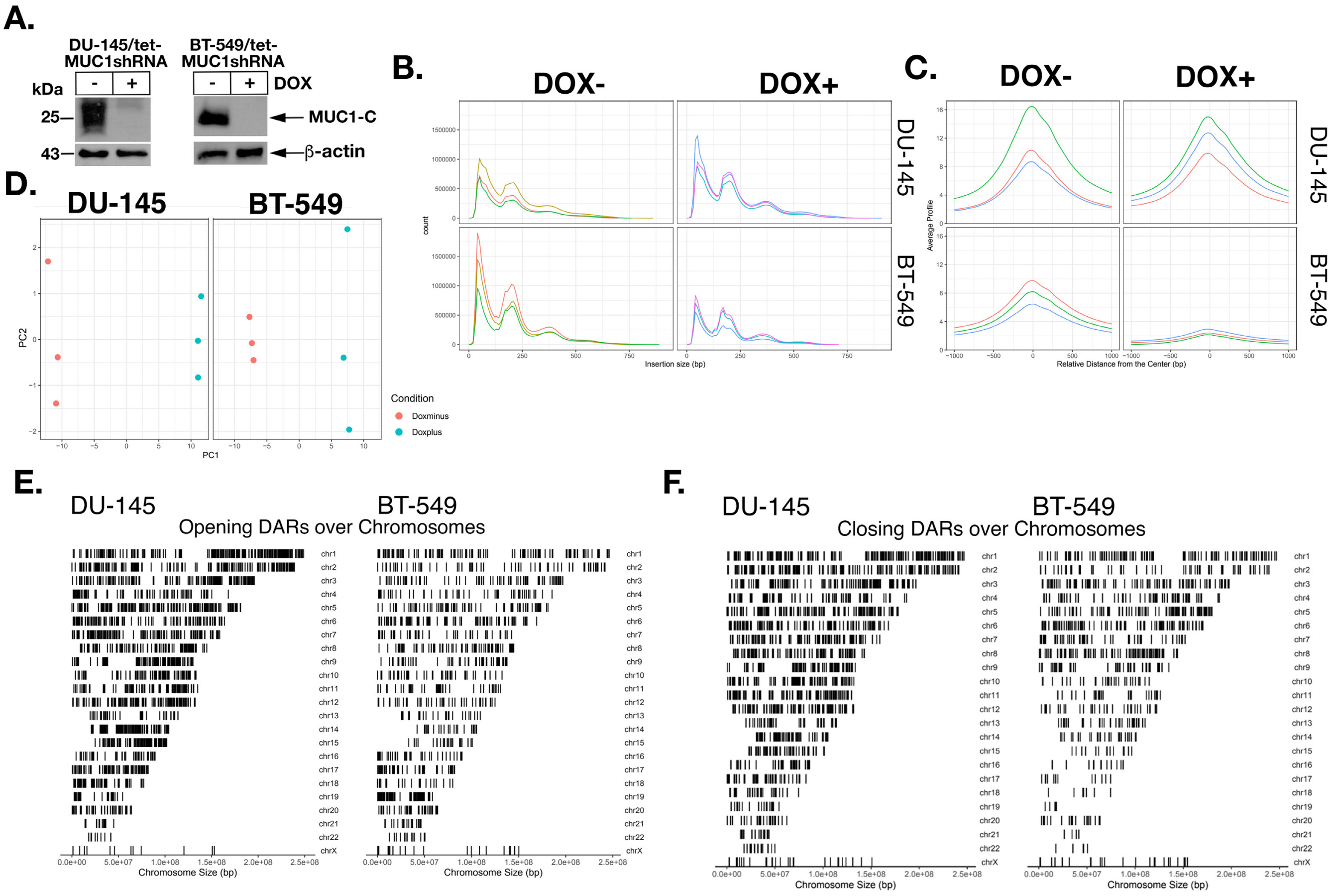

Supplemental Figure S1. Analysis of ATAC-seq libraries. A. DU-145/tet-MUC1shRNA (left) and BT-549/tet-MUC1shRNA (right) cells were treated with vehicle or DOX for 7 days. Lysates were immunoblotted with the indicated antibodies. **B.** Fragment size distribution of ATAC-seq libraries showing phasing of mono-, di- and tri-nucleosomal regions. **C.** Presentation of ATAC-seq read distributions as an average plot depicting signal enrichment at gene start sites. **D.** Principal Component Analysis (PCA) depicting data variability and clustering of samples by group. **E and F.** Chromosomal localization of opening (**E**) and closing (**F**) DARs in MUC1-C-silenced DU-145 (left) and BT-549 (right) cells.

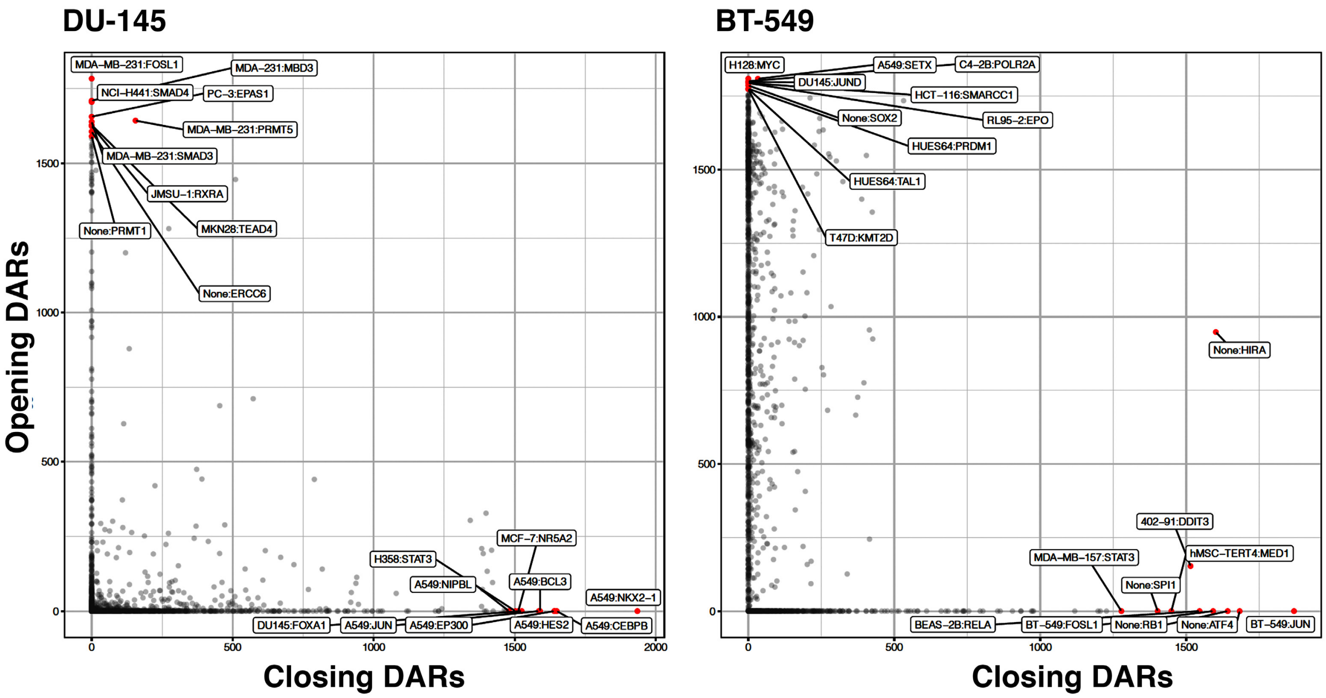

Supplemental Figure S2. Cistromes in MUC1-C-induced DARs. DARs from DU-145 (left) and BT-549 (right) were queried against all publicly available ChIP-seq datasets collected in the Cistrome DB. The x and y-axis show the giggle-scores measuring the significance of overlaps between either opening or closing DARs and the top 1000 peaks from each ChIP-seq dataset. The red dots highlight the top10 hits for either opening or closing DARs.

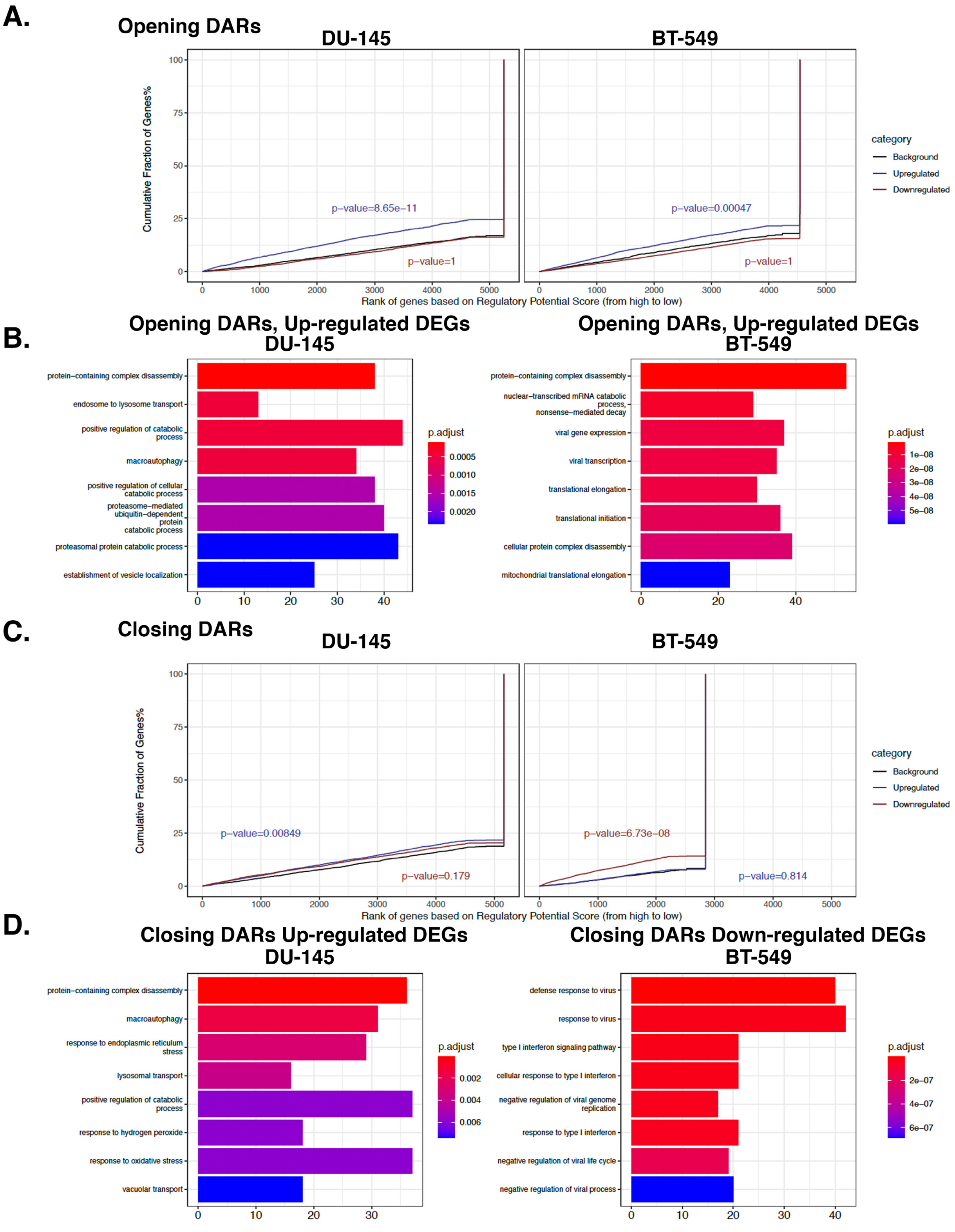

E.

DU-145	LZ-TF	T-test p-value
MC00456	JUND	3.46e-05
MC00321	JUN	6.33e-04
MC00371	JUNB	1.50e-03
MS00336	NFE2	1.41e-03
MC00351	FOSL1	9.46e-04
MC00330	FOS	3.33e-03

BT-549	LZ-TF	T-test p-value
MC00456	JUND	4.03e-08
MC00321	JUN	6.45e-07
MC00371	JUNB	7.48e-08
MS00336	NFE2	1.21e-04
MC00351	FOSL1	8.95e-07
MC00330	FOS	7.54e-07

Supplemental Figure S3. Associations of DARs and corresponding DEGs with GO BIOLOGICAL PROCESSES. A. Correlations of opening DARs with up- and down-regulated DEGs in DU-145 (left) and BT-549 (right) cells. **B.** Associations of opening DARs and upregulated DEGs with GO BIOLOGICAL PROCESSES in DU-145 (left) and BT-549 (right) cells. **C.** Correlations of closing DARs with up- and down-regulated DEGs in DU-145 (left) and BT-549 (right) cells. **D.** Associations of closing DARs and the indicated DEGs with GO BIOLOGICAL PROCESSES in DU-145 (left) and BT-549 (right) cells. E. Associations of DARs and DEGs with motifs recognized by the indicated AP-1 family members in DU-145 (left) and BT-549 (right) cells.

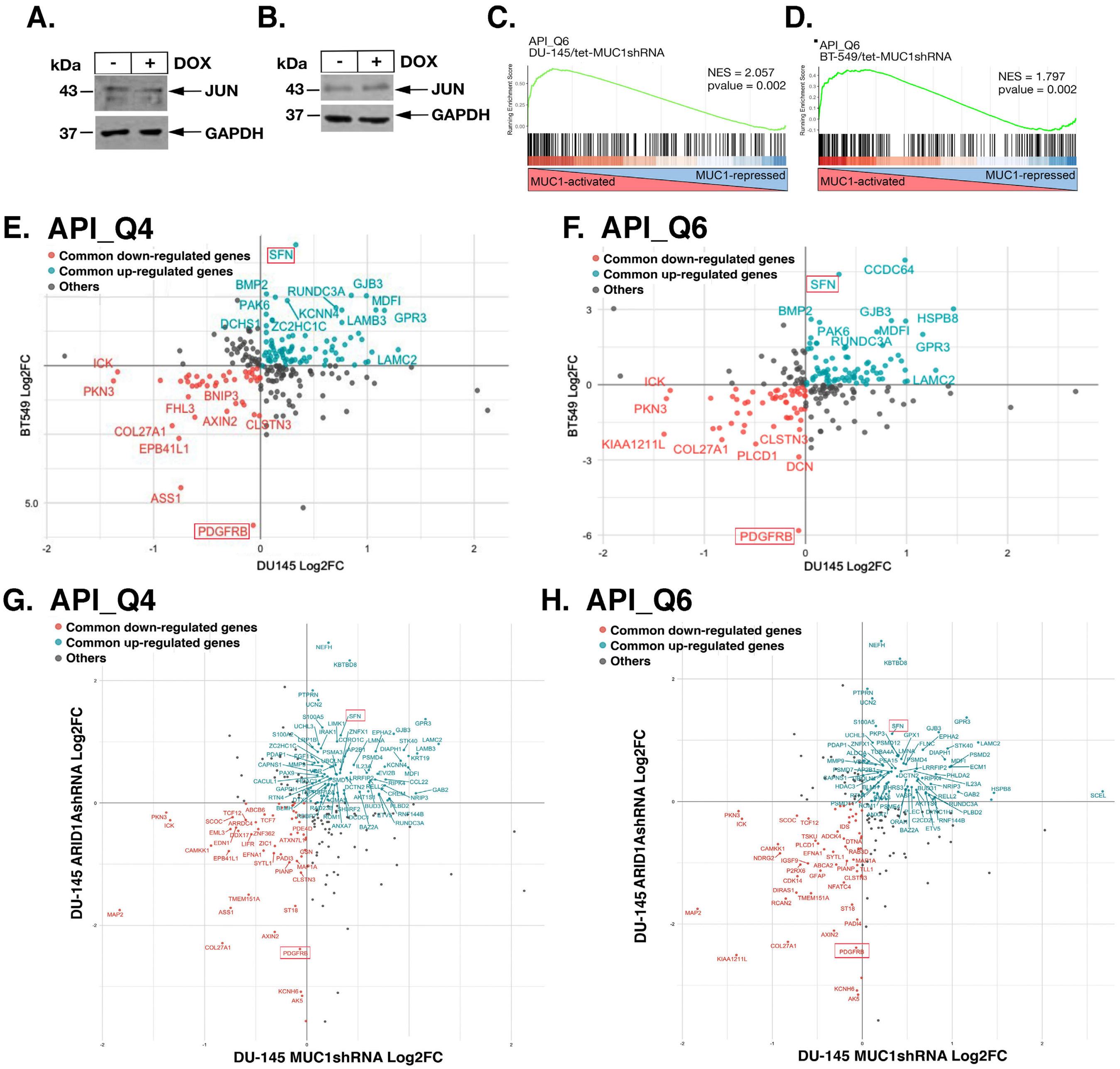

Supplemental Figure S4. Associations of MUC1 and JUN/AP-1 gene signatures. A and B. Lysates from DU-145/tet-MUC1shRNA cells (**A**) and BT-549/tet-MUC1shRNA (**B**) cells treated with vehicle or DOX for 7 days were immunoblotted with antibodies against the indicated proteins. **C and D.** RNA-seq was performed in triplicate on DU-145/tet-MUC1shRNA (**C**) and BT-549/tet-MUC1shRNA (**D**) cells treated with vehicle or DOX for 7 days. The datasets were analyzed with GSEA using the AP-1 Q6 gene signature. **E and F.** Overlap of down- and up-regulated genes in DU-145 and BT-549 cells with MUC1-C silencing obtained from GSEA of the AP-1 Q4 (**E**) and AP-1 Q6 (**F**) target gene signatures. **G and H.** Overlap of down- and up-regulated genes in DU-145 cells with MUC1-C and ARID1A silencing obtained from GSEA of the AP-1 Q4 (**G**) and AP-1 Q6 (**H**) target gene signatures.

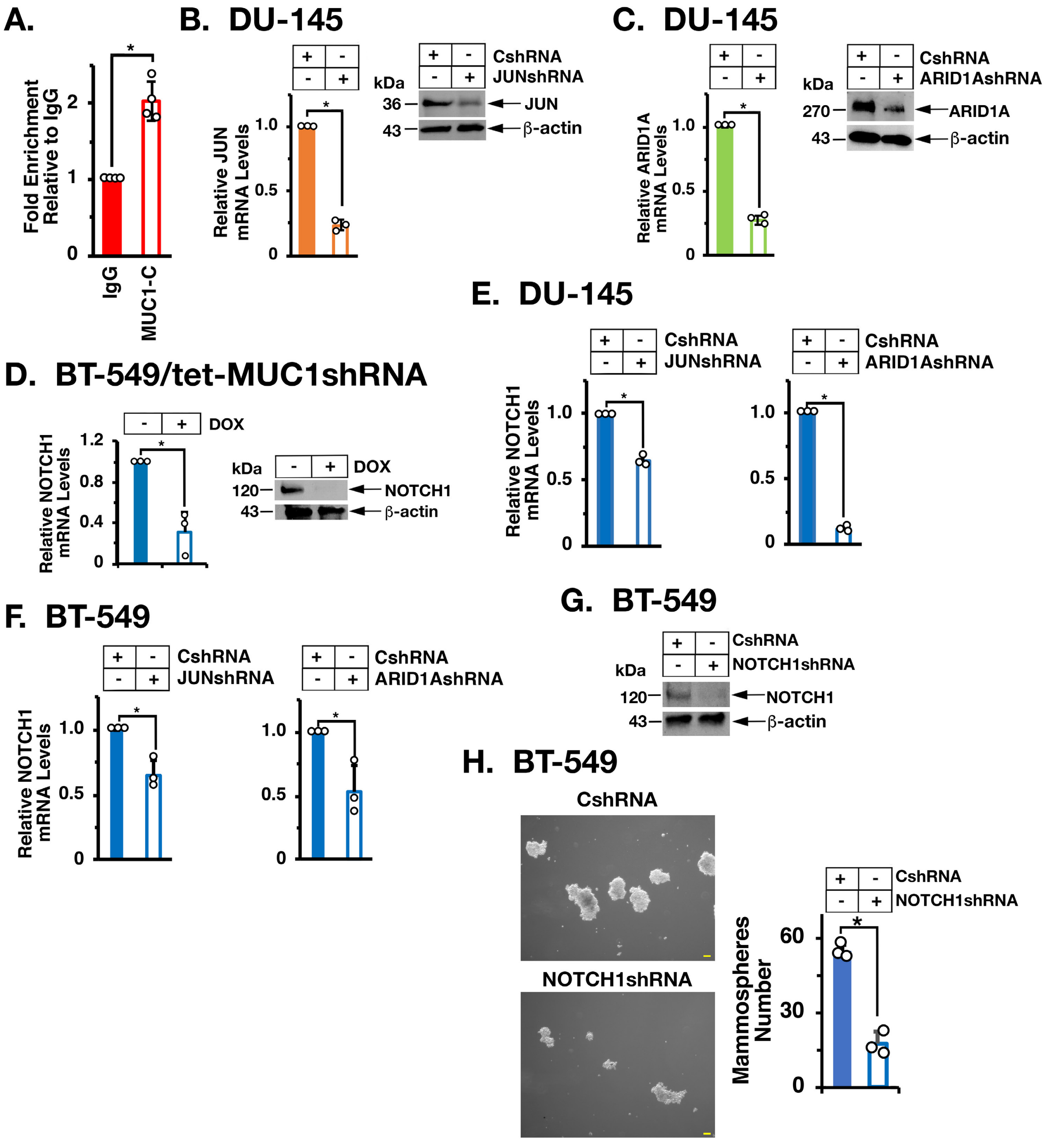

Supplemental Figure S5. MUC1-C forms a complex with JUN and effects of silencing MUC1-C, JUN and ARID1A. A. Soluble chromatin from DU-145 cells was precipitated with a control IgG or anti-JUN (ChIP) and then reprecipitated with a control IgG or anti-MUC1-C (Re-ChIP). The DNA samples were amplified by qPCR with primers for the *NOTCH1* PELS. The results (mean±SD of 3 determinations) are expressed as fold enrichment relative to that obtained with the IgG control (assigned a value of 1). **B.** DU-145 cells expressing a CshRNA or JUNshRNA were analyzed for JUN mRNA levels by qRT-PCR (left). The results (mean±SD of

3 determinations) are expressed as relative mRNA levels compared to that obtained for the CshRNA cells (assigned a value of 1). Lysates were immunoblotted with antibodies against the indicated proteins (right). **C.** DU-145 cells expressing a CshRNA or ARID1AshRNA were analyzed for ARID1A mRNA levels by qRT-PCR (left). The results (mean±SD of 3 determinations) are expressed as relative mRNA levels compared to that obtained for the CshRNA cells (assigned a value of 1). Lysates were immunoblotted with antibodies against the indicated proteins (right). **D.** BT-549/tet-MUC1shRNA cells treated with vehicle or DOX for 7 days were analyzed for NOTCH1 mRNA levels by qRT-PCR (left). The results (mean±SD of 3 determinations) are expressed as relative mRNA levels compared to that obtained for control cells (assigned a value of 1). Lysates were immunoblotted with antibodies against the indicated proteins (right). **E.** DU-145/CshRNA, DU-145/JUNshRNA and DU-145/ARID1AshRNA cells were analyzed for NOTCH1 mRNA levels by qRT-PCR. **F.** BT-549/CshRNA, BT-549/JUNshRNA and BT-549/ARID1AshRNA cells were analyzed for NOTCH1 mRNA levels by qRT-PCR. The results (mean±SD of 3 determinations) are expressed as relative mRNA levels compared to that obtained for CshRNA cells (assigned a value of 1). **G.** Lysates from BT-549/CshRNA and BT-549/NOTCH1shRNA cells were immunoblotted with antibodies against the indicated proteins. **H.** BT-549/CshRNA and BT-549/NOTCH1shRNA cells (5000/well) were assayed for tumorsphere formation at 10 days (left). Scale bar: 100 um. The results (mean+SD of 3 biological replicates) are expressed as the number of mammospheres (right).

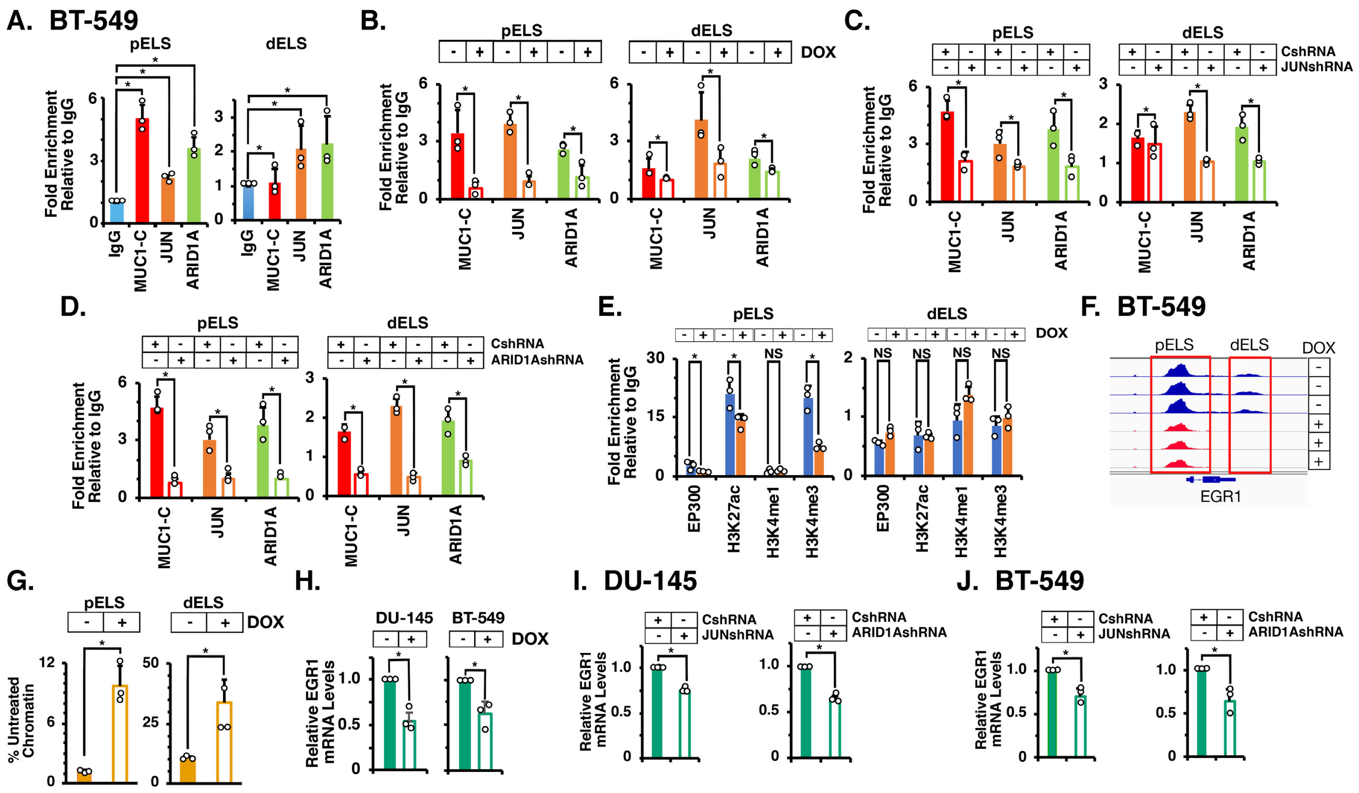

Supplemental Figure S6. MUC1-C activates the *EGR1* pELS and dELS in BT-549 cells. A. Soluble chromatin from BT-549 cells was precipitated with a control IgG, anti-MUC1-C, anti-JUN, anti-ARID1A and anti-PBRM1. **B.** Soluble chromatin from BT-549/tet-MUC1shRNA cells treated with vehicle or DOX was precipitated with a control IgG, anti-MUC1-C, anti-JUN and anti-ARID1A. **C.** Soluble chromatin from BT-549/CshRNA and BT-549/JUNshRNA cells was precipitated with a control IgG, anti-MUC1-C, anti-JUN and anti-ARID1A. **D.** Soluble chromatin from BT-549/CshRNA and BT-549/ARID1AshRNA cells was precipitated with a control IgG, anti-MUC1-C, anti-JUN and anti-ARID1A. **E.** Soluble chromatin from BT-549/tet-MUC1shRNA cells treated with vehicle or DOX was precipitated with a control IgG, anti-EP300, anti-H3K27ac, anti-H3K4me1 and anti-H3K4me3. The DNA samples were amplified by qPCR with primers for the *EGR1* pELS (left) and dELS (right). The results (mean±SD of 3 determinations) are expressed as fold enrichment relative to that obtained with the IgG control (assigned a value of 1). **F and G.** BT-549/tet-MUC1shRNA cells were treated with vehicle or DOX for 7 days. Genome browser snapshots of ATAC-seq data from the *EGR1* pELS and dELS (**F**). Chromatin was analyzed for accessibility by nuclease digestion (**G**). The results (mean±SD of 3 determinations) are expressed as % untreated chromatin. **H.** DU-145/tet-MUC1shRNA (left) and BT-549/tet-MUC1shRNA (right) cells treated with vehicle or DOX for 7 days were analyzed for EGR1 mRNA levels by qRT-PCR. **I and J.** The indicated DU-145 (**I**) and BT-549 (**J**) cells expressing a CshRNA, JUNshRNA or ARID1AshRNA were analyzed for EGR1 mRNA levels by qRT-PCR. The results (mean±SD of 3 determinations) are expressed as relative mRNA levels compared to that obtained for control cells (assigned a value of 1).

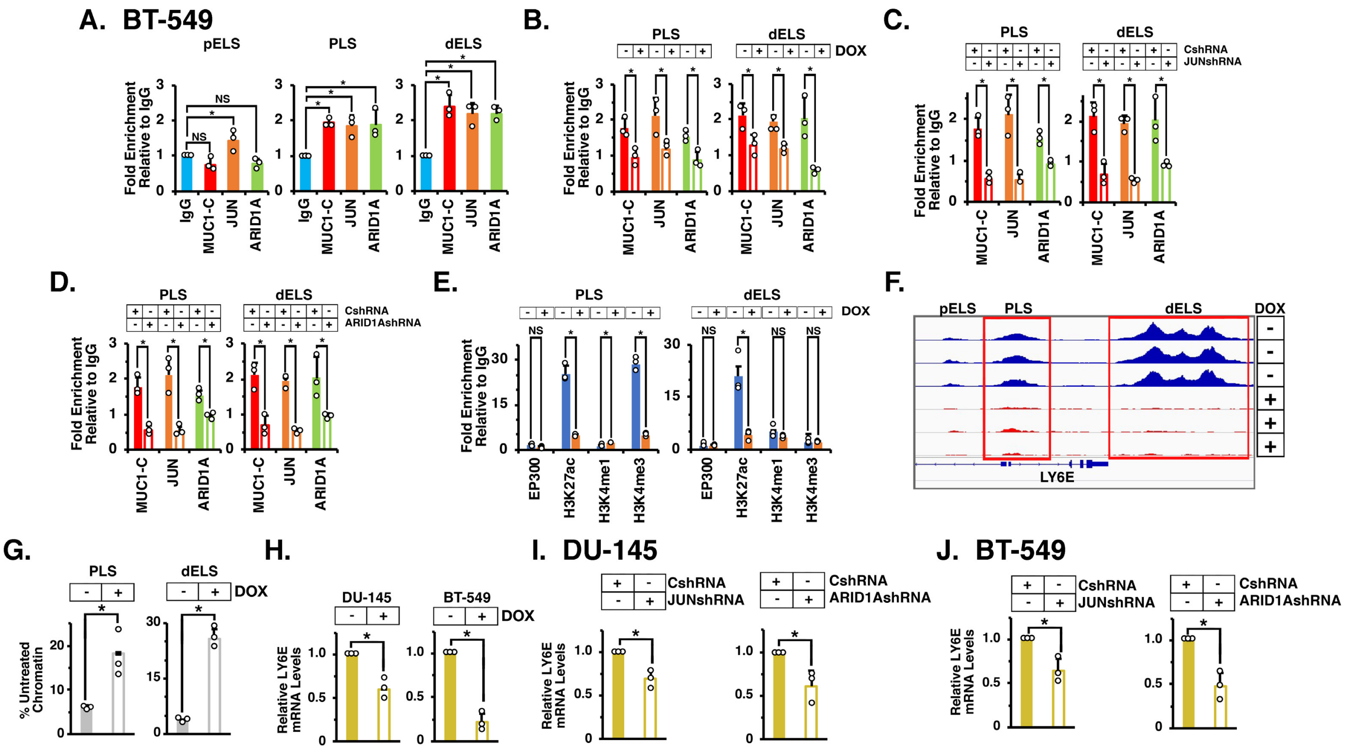

Supplemental Figure S7. MUC1-C activates the *LY6E* pELS and dELS in BT-549 cells. A. Soluble chromatin from BT-549 cells was precipitated with a control IgG, anti-MUC1-C, anti-JUN and anti-ARID1A. The DNA samples were amplified by qPCR with primers for the *LY6E* pELS (left), PLS (middle) and dELS (right). **B.** Soluble chromatin from BT-549/tet-MUC1shRNA cells treated with vehicle or DOX was precipitated with a control IgG, anti-MUC1-C, anti-JUN and anti-ARID1A. **C.** Soluble chromatin from BT-549/CshRNA and BT-549/JUNshRNA cells was precipitated with a control IgG, anti-MUC1-C, anti-JUN and anti-ARID1A. **D.** Soluble chromatin from BT-549/CshRNA and BT-549/ARID1AshRNA cells was precipitated with a control IgG, anti-MUC1-C, anti-JUN and anti-ARID1A. **E.** Soluble chromatin from BT-549/tet-MUC1shRNA cells treated with vehicle or DOX was precipitated with a control IgG, anti-EP300, anti-H3K27ac, anti-H3K4me1 and anti-H3K4me3. The DNA samples were amplified by qPCR with primers for the *LY6E* PLS (left) and dELS (right). The results (mean±SD of 3 determinations) are expressed as fold enrichment relative to that obtained with the IgG control (assigned a value of 1). **F and G.** BT-549/tet-MUC1shRNA cells were treated with vehicle or DOX for 7 days. Genome browser snapshots of ATAC-seq data from the *LY6E* pELS, PLS and dELS (**F**). Chromatin from the PLS and dELS was analyzed for accessibility by nuclease digestion (**G**). The results (mean±SD of 3 determinations) are expressed as % untreated chromatin. **H.** DU-145/tet-MUC1shRNA (left) and BT-549/tet-MUC1shRNA (right) cells treated with vehicle or DOX for 7 days were analyzed for LY6E mRNA levels by qRT-PCR. **I.** DU-145/CshRNA, DU-145/JUNshRNA and DU-145/ARID1AshRNA cells were analyzed for LY6E mRNA levels by qRT-PCR. **J.** BT-549/CshRNA, BT-549/JUNshRNA and BT-549/ARID1AshRNA cells were analyzed for LY6E mRNA levels by qRT-PCR. The results (mean±SD of 3 determinations) are expressed as relative

mRNA levels compared to that obtained for CshRNA cells (assigned a value of 1).

Table S1. Primers used for qRT-PCR.

**NOTCH1**	FWD	GGGCTAACAAAGATATGCAG
	REV	ACTGAACCTGACCGTACAGTTGGCAAAGTGGTCCAG
**JUN**	FWD	CCAAAGGATAGTGCGATGTTT
	REV	CTGTCCCTCTCCACTGCAAC
**GAPDH**	FWD	CCATGGAGAAGGCTGGGG
	REV	CAAAGTTGTCATGGATGACC
**EGR1**	FWD	CTTCAACCCTCAGGCGGACA
	REV	GGAAAAGCGGCCAGTATAGGT
**ARID1A**	FWD	ACCTCTATCGCCTCTATGTGTCTGT
	REV	CTGGCAGCACTGCTTGATGT
**LY6E**	FWD	CTCCAGGCAGGACGGCCATC
	REV	CGAGATTCCCAATGCCGGCACT

Table S2. Primers used for direct chromatin accessibility assays.

**NOTCH1 pELS**	FWD	CCTGGGACTACTTCTCGTTTG
	REV	GCAAATTTCAGTCGCCAGTTG
**EGR1 pELS**	FWD	ATTCAGAGCTAGAGCAGGAGGAG
	REV	GGTGGCGAGGGAGAACGATT
**EGR1 dELS**	FWD	AAGTGCTGGGATTACAGGC
	REV	CAAAGTATGACCCTCCCATCTC
**LY6E pELS**	FWD	ATGTGTTTCCCTGAGTTCCC
	REV	ACCCTCTTTCCCAGCAATAC
**LY6E PLS**	FWD	GGAAGCAGGGACAAGATGAC
	REV	ACGTGTTTGGGTGTGAGC
**LY6E dELS**	FWD	CTTCATGGTCTTGGGTATGGG
	REV	AACAATCCGGGTTCCTCATC

Table S3. Primers used for ChIP-qPCR.

**NOTCH1 pELS**	FWD	CCTGGGACTACTTCTCGTTTG
	REV	GCAAATTTCAGTCGCCAGTTG
**pGAPDH**	FWD	TACTAGCGGTTTTACGGGCG
	REV	TCGAACAGGAGGAGCAGAGAGCGA
**EGR1 pELS**	FWD	ATTCAGAGCTAGAGCAGGAGGAG
	REV	GGTGGCGAGGGAGAACGATT
**EGR1 dELS**	FWD	AAGTGCTGGGATTACAGGC
	REV	CAAAGTATGACCCTCCCATCTC
**LY6E pELS**	FWD	ATGTGTTTCCCTGAGTTCCC
	REV	ACCCTCTTTCCCAGCAATAC
**LY6E PLS**	FWD	GGAAGCAGGGACAAGATGAC
	REV	ACGTGTTTGGGTGTGAGC
**LY6E dELS**	FWD	CTTCATGGTCTTGGGTATGGG
	REV	AACAATCCGGGTTCCTCATC
